# Monitoring the photochemistry of a formazan over 15 orders of magnitude in time

**DOI:** 10.3389/fchem.2022.983342

**Published:** 2022-09-28

**Authors:** Svenja Wortmann, Roger Jan Kutta, Patrick Nuernberger

**Affiliations:** Institut für Physikalische und Theoretische Chemie, Universität Regensburg, Regensburg, Germany

**Keywords:** formazan, photoisomerization, transient absorption, femtochemistry, streak camera, excited-state dynamics, proton transfer, ground-state dynamics

## Abstract

2,3,5-triphenyltetrazolium chloride (TTC) may convert into phenyl-benzo[c]tetrazolocinnolium chloride (PTC) and 1,3,5-triphenylformazan (TPF) under irradiation with light. The latter reaction, albeit enzymatically rather than photochemically, is used in so-called TTC assays indicating cellular respiration and cell growth. In this paper, we address the photochemistry of TPF with time-resolved spectroscopy on various time scales. TPF is stabilized by an intramolecular hydrogen bond and switches photochemically *via* an E-Z isomerization around an N=N double bond into another TPF-stereoisomer, from which further isomerizations around the C=N double bond of the phenylhydrazone group are possible. We investigate the underlying processes by time-resolved spectroscopy in dependence on excitation wavelength and solvent environment, resolving several intermediates over a temporal range spanning 15 orders of magnitude (hundreds of femtoseconds to hundreds of seconds) along the reaction path. In a quantum-chemical analysis, we identify 16 stable ground-state isomers and discuss which ones are identified in the experimental data. We derive a detailed scheme how these species are thermally and photochemically interconnected and conclude that proton transfer processes are involved.

## 1 Introduction

**FIGURE 1:**
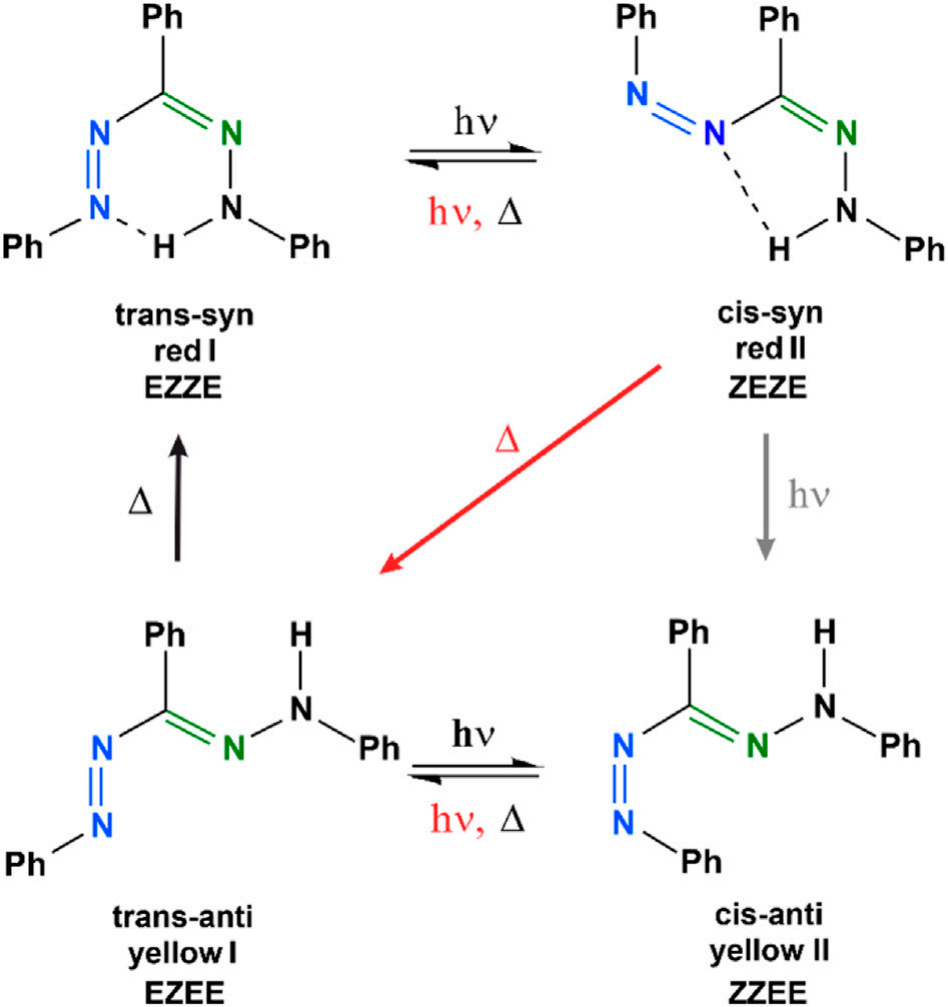
The two most prominent photoisomerization schemes of TPF after excitation, as introduced by Kuhn and Weitz (Kuhn and Weitz, 1953) and by Grummt and Langbein (Grummt and Langbein, 1981). Black arrows and symbols indicate pathways present in both models, while gray elements are only found in the Kuhn-Weitz scheme and red elements only in the Grummt-Langbein scheme, respectively. The nomenclature, e.g., trans-syn, corresponds to the N=N double bond (blue) and the C=N double bond (green). The orientation of the adjacent C–N and N–N single bonds will be discussed in Section 4.

Formazans comprise an azo group (–N=N–) and a hydrazone group (–C=N–N–), with both being capable to isomerize at the double bond upon excitation with visible light. The most investigated representative is 1,3,5-triphenylformazan (TPF, Figure 1). TPF can be generated by a photochemical conversion of 2,3,5-triphenyltetrazolium chloride (TTC), accompanied by a color change of the solution from colorless to red (Hausser et al., 1949a; Grummt and Langbein, 1981). TPF exhibits a unique photochromism which depends on the excitation conditions and the solvent environment. We investigated in an earlier study to which extent the thermal back relaxation around the C=N double bond is sensitive to the polarity and the hydrogen-bond donating ability of the solvent (Wortmann et al., 2022). While polar solvents with a higher polarity result in a decrease of the activation barrier of the anti-syn isomerization around the C=N bond, hydrogen bonding has an oppositely directed effect and can stabilize the trans-anti isomer under certain conditions. Due to its distinct photochromism and this high sensitivity toward external influences, but mostly because of its enzymatic formation from TTC, TPF is found in a wide field of applications. These are found in biological assays to indicate cellular respiration and cell growth (Ziegler, 1953; Mosmann, 1983; Berridge et al., 2005), in agriculture to verify the germinability of seeds (Lakon, 1942; Smith, 1951), in medicine, especially in cancer research (Carmichael et al., 1987; Alley et al., 1988; Scudiere et al., 1988), but also in areas like dosimetry (Kovács et al., 1996), chemical synthesis (Neugebauer and Russell, 1968; Lipunova et al., 2019), or as chelating agents in organometallic chemistry (Lipunova et al., 2019; Gilroy and Otten, 2020). In general, most of these applications rely on the reduction of the colorless tetrazolium salt TTC to TPF, so that a colorless solution turns red (Hausser et al., 1949a). The photochemistry of the precursor TTC has already been explored in detail with various techniques (Pechmann and Runge, 1894; Hausser et al., 1949b; Nineham, 1955; Neugebauer and Russell, 1968; Umemoto, 1985; Gonzalez and San Roman, 1989; Kovács et al., 1996; Kanal et al., 2015; Bolze et al., 2018). Despite several studies with a focus on quantum-chemical calculations (Buemi et al., 1998; Buemi and Zuccarello, 2002; King and Murrin, 2004; Tezcan and Tokay, 2010; Sherif, 2015), luminescence (Turkoglu et al., 2015), solvatochromism (Sherif, 1997; Kumar et al., 2014; Wortmann et al., 2022), Raman and infrared studies (Schiele, 1965; Otting and Neugebauer, 1968; Otting and Neugebauer, 1969; Lewis and Sandorfy, 1983; Hiura and Takahashi, 1989a; Hiura and Takahashi, 1989b; Tezcan and Ozkan, 2003), or electrochemical properties (Gökçe et al., 2005; Sherif, 2015; Turkoglu et al., 2015) of formazans, also investigated under temperature (Kuhn and Weitz, 1953; Langbein, 1979; Grummt and Langbein, 1981; Sueishi and Nishimura, 1983) or pressure variation (Sueishi and Nishimura, 1983), the interplay of light-induced processes which set in on an ultrafast time scale and extend to minutes has not been comprehensively studied for TPF. With regard to the involved intermediate species of TPF, several photoisomerization mechanisms of TPF are discussed in literature, with the one proposed by Grummt and Langbein which was derived from laser flash photolysis experiments (Grummt and Langbein, 1981) being in closest accordance to the one inferred in our recent study (Wortmann et al., 2022). Note that also the orientation of the single bonds adjacent to the double bonds are sometimes drawn differently (Lewis and Sandorfy, 1983; Veas-Arancibia, 1986) and lead to further stable isomers, as we will discuss in detail in Section 4. The energetically most-stable isomer is the trans-syn form (also called “red I”, Figure 1), which is stabilized by an intramolecular hydrogen bond, forming a quasi-aromatic heterocycle. Illumination with visible light leads to an isomerization around the N=N double bond, yielding a cis-syn isomer (“red II”, Figure 1). For this species, an intramolecular hydrogen bonding can occur as well. Afterwards, thermal isomerization around both N=N and C=N leads to the trans-anti analogue (“yellow I”, Figure 1), which is accompanied by a color change of the solution from red to yellow. Spectroscopically, a hypsochromic shift from around 490 nm to 405 nm is observable in toluene solutions (Kuhn and Weitz, 1953; Langbein, 1979; Grummt and Langbein, 1981; Atabekyan et al., 2011; Wortmann et al., 2022). Under dark conditions, the trans-anti isomer relaxes back into the energetically-favored trans-syn form *via* an anti-syn isomerization around the C=N double bond. However, when absorbing visible light, the trans-anti isomer may follow another pathway, yielding a second yellow species, the cis-anti analogue (“yellow II”, Figure 1). Both yellow forms can be characterized by absorption spectra with different extinction coefficients (Hausser et al., 1949a; Langbein, 1979; Grummt and Langbein, 1981) and a broader absorption band for yellow II.

Here, we address three aspects of the light-induced reactions of TPF. First, we want to monitor all relevant time scales, from the primary steps occurring in sub-ps to the slowest thermal equilibrations occurring within minutes. Several different pump-probe setups were used to monitor this extended time window, that is fs to ns transient absorption (TA) based on femtosecond lasers, ns to ms TA with ns excitation pulses, and ms up to minutes with pulsed light-emitting diodes (LEDs) as pump source. Second, we reassess the assignment of the involved isomers by DFT and TD-DFT calculations. Third, we discuss the role of proton transfer to the thermal interconversion of the isomers.

## 2 Materials and methods

TPF was purchased from Sigma-Aldrich, used without further purification, and dissolved in anhydrous acetonitrile (Sigma-Aldrich) or methanol (Uvasol, Merck) of spectroscopic grade. UV-Vis absorption spectra were recorded on a spectrophotometer (Cary60, Agilent) which could be combined with LED excitation in a perpendicular arrangement in order to record ms to min TA data with a time increment of 12.5 ms. For some of the employed LEDs, a different spectrophotometer (UV 1800, Shimadzu) was employed for the same purpose, with a time increment of 480 ms. The LED temporal rectangular pulse width was also set to 12.5 or 480 ms, respectively. The sample (250 μL) was inside a rectangular quartz cuvette (Starna, 10 mm × 2 mm) and the absorption was monitored over the 10 mm optical path length, while the LED was adjusted to illuminate the entire sample, thus aiming at a homogeneous illumination over the 2 mm path length.

For ns to ms TA, a Nd:YAG laser (Surelite II, Continuum) in combination with an optical parametric oscillator (Surelite OPO Plus, Continuum) generated the pump pulses, while the broadband probe light originated from a Xenon flash lamp (MSP-05, Müller Elektronik-Optik). A streak camera (C7700, Hamamatsu Photonics) with spectrograph and CCD camera (ORCA-CR, Hamamatsu Photonics) was employed for detection [see (Kutta et al., 2013; Dick et al., 2019) for further details on the experimental implementation]. The sample was circulated through the 2 mm (excitation path) × 10 mm (probe path) quartz cuvette from an external reservoir of 4 mL.

For fs to ns TA, a Ti:Sa amplifier system (Libra, Coherent) generated laser pulses at a repetition rate of 1 kHz centered at 800 nm that were used to pump an optical parametric amplifier (TOPAS-C, Light Conversion), yielding pulses at 530 nm or (after a further nonlinear process) 330 nm used for excitation of the sample. For the probe beam, parts of the 800 nm were used to pump a home-built noncollinear optical parametric amplifier adjusted to ca. 500 nm that then was focused into a moving calcium fluoride plate generating a white-light supercontinuum (Dobryakov et al., 2010). This was split into a reference bypassing the sample and a probe beam traversing the sample. These two beams were independently imaged onto two home-built grating spectrographs, and their spectra were recorded with photodiode arrays (S3901-512Q, Hamamatsu, 512 pixels) at 1.5 nm resolution. The polarizations of pump and probe beams were set to the magic angle (Megerle et al., 2009; Schott et al., 2014) before reaching the 2 mm × 10 mm quartz cuvette. The temporal resolution was around 100 fs, and the temporal delay was introduced by a cornercube retroreflector on a delay stage (M-531.2S, Physik Instrumente) placed in the pump beam. More details on the employed setup are given in (Brandl et al., 2020).

Quantum-mechanical calculations on all stable TPF conformers were performed using the Orca package (Neese, 2012; Neese, 2018). All ground-state structures were optimized on the level of restricted closed shell density functional theory (RHF-DFT) using the B3LYP functional and the def2-TZVP basis set with D4 dispersion correction. Potential barriers connecting the individual conformers were roughly estimated (as DFT is not able to correctly describe the bond rotation around double bonds) from the crossing points for the relaxed potential energy surfaces along the rotational motion around a corresponding bond starting from each stable conformer using B3LYP/def2-TZVP level of theory.

## 3 Experimental results and discussion

**FIGURE 2:**
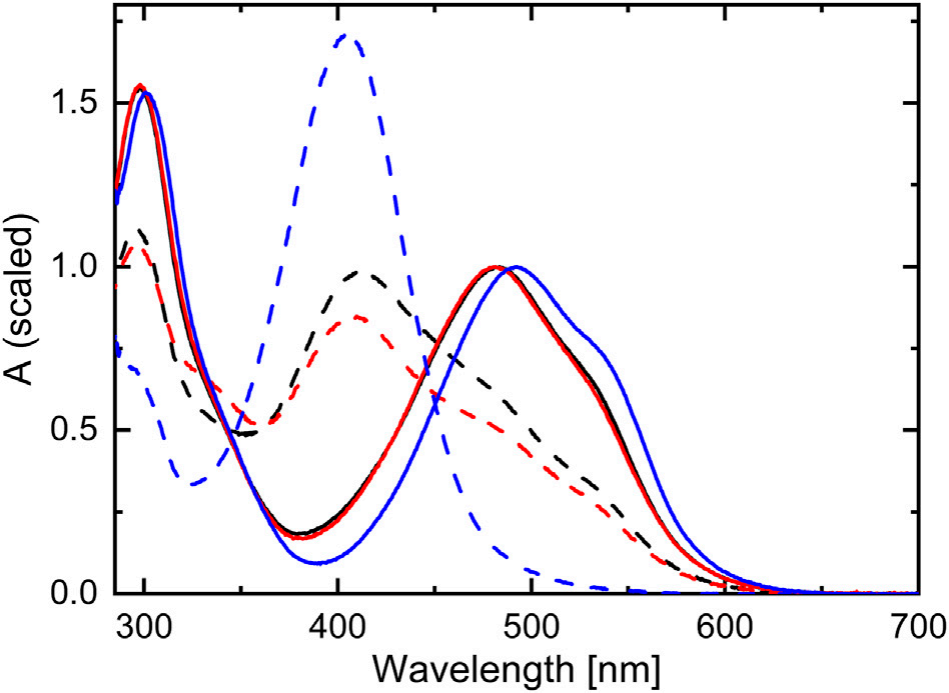
Steady-state absorption spectra of TPF dissolved in methanol (black), acetonitrile (red), and toluene (blue). The solid curves display the ground-state absorption spectra (normalized to the maximum around 500 nm), while the dashed spectra correspond to the photostationary state under illumination with 520 nm light.

The steady-state UV-Vis absorption spectra of TPF detected in methanol and acetonitrile solution are nearly identical, with two main absorption bands peaking around 300 and 480 nm, of which the latter comprises a weak shoulder on its red edge (see Figure 2). In unsubstituted formazan, the lowest electronic transition is of $n-\pi^{*}$ character (Buemi et al., 1998). The same is found for TPF, albeit with a negligible oscillator strength, so that the major absorption band in the visible is dominated by a $\pi -\pi^{*}$ transition (Avramenko and Stepanov, 1974; Nădejde et al., 2009) to the second excited state S_2_ (see also Supplementary Figure S6 with corresponding DFT calculations). For the TPF absorption spectrum in toluene solution, a small spectral shift of the low-frequency $\pi -\pi^{*}$ transition band by ∼10 nm is observed. Under irradiation with visible light, the formation of the yellow isomeric species was reported to occur in acetonitrile (Wilhite, 1991) as well as in toluene/benzene solutions (Kuhn and Weitz, 1953; Langbein, 1979; Grummt and Langbein, 1981; Atabekyan et al., 2011) and is manifested by an increased absorption around 400 nm. Constantly illuminating TPF in methanol solution with 520 nm light also leads to the formation of the yellow species (Figure 2). In comparison to toluene, where the equilibrium is nearly fully shifted under these conditions, in acetonitrile and methanol a distinct amount of TPF molecules is observed in its initial conformation, indicated by both the lower absorption around 400 nm (yellow I) and the remaining contribution at 500 nm (compare black/red dashed curves in Figure 2 with the blue dashed curve).

### 3.1 fs-ns transient absorption of 1,3,5-triphenylformazan

**FIGURE 3:**
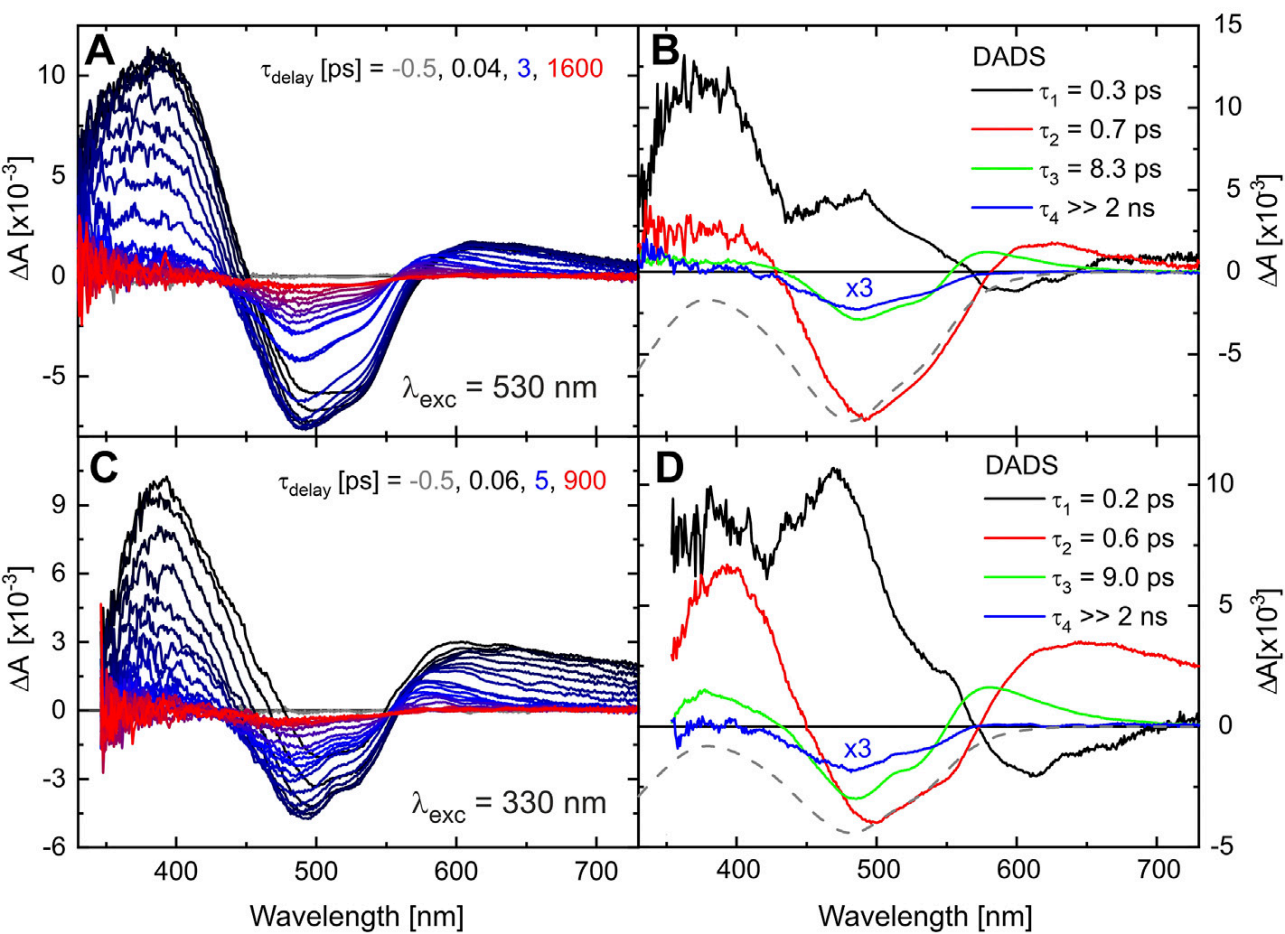
Transient absorption of TPF in methanol after excitation at 530 nm **(A)** and 330 nm **(C)** after defined delay times on a fs-ns time scale. The corresponding DADS from a global exponential fit to the data are given in **(B,D)**. The scaled and inverted absorption spectrum of the initial sample is given by a gray-dashed line for comparison.

The initial photodynamics of TPF were followed by recording the TA on a fs to ns time scale after excitation either at 530 or 330 nm. Transient absorption spectra of TPF are shown in Figures 3A,C for methanol solution. In both cases, two regions of increased absorption around 400 and 600 nm are observed, together with the ground-state bleach (GSB) in the spectral region around 500 nm. Whereas the overall signal intensity decreases with time, a contribution persists beyond the experimental time window of 2 ns (Kanal et al., 2015). The decay-associated difference spectra (DADS), resulting from a global multiexponential fit to the data matrices with four time constants, are displayed in Figures 3B,D, respectively [contributions for modelling the coherent artefact (Megerle et al., 2009) are not shown for clarity].

The fastest process, with decay time $\tau_{1}$ being 0.3 and 0.2 ps, respectively, for the two excitation wavelengths, may be assigned to isomerization dynamics connected to the azo group. For trans-azobenzene, the excited-state dynamics directly towards a conical intersection exhibit a decay time around 0.3 ps (Nägele et al., 1997; Lu et al., 2002; Satzger et al., 2003; Satzger et al., 2004; Pancur et al., 2005; Quick et al., 2014; Nenov et al., 2018), with similar values reported for related compounds (Siewertsen et al., 2011; Slavov et al., 2016; Grimmelsmann et al., 2017). However, there is also a further excited-state motion in trans-azobenzene on a time scale of 1–3 ps, assigned to diffusion-type motion (Nägele et al., 1997) or the passage of a barrier in the excited state (Quick et al., 2014). Along these lines, the positive signals around 400 and 600 nm (red DADS in Figure 3B,D) may indicate absorption features of molecules still in an excited state that depopulates with decay time $\tau_{2}$ mainly into the ground state configuration that was present prior to excitation, as can be seen by the negative contribution in the red DADS. We link the more pronounced excited state absorption (ESA) for excitation at 330 nm compared to 530 nm to the excess energy introduced by the higher photon energy. The DADS with $\tau_{3}$ (green DADS) may reflect relaxation dynamics accompanied by vibrational cooling of molecules either in the excited state or already in the ground state all ending in the most stable conformer that was initially excited. Although the dominant fraction of excited molecules returns to the initial ground state configuration, a GSB at 480 nm and a product absorption around 330 nm remains on a time scale > 2 ns (see also blue DADS) substantiating the formation of another stable photoisomer. Note that TPF is in an equilibrium of two ground state conformers with predominantly red I but also a few red II conformers, so that the TA data observed after excitation at 330 nm resemble the superposed dynamics of both isomers which explains the small differences in the absorption strength and dynamics compared to the situation when exciting at 530 nm. This aspect will become more evident on a longer time scale (vide infra).

The TA data in the fs-ns time range of TPF in the solvent acetonitrile are very similar and can be analyzed and interpreted accordingly (see Supplementary Figure S1). Only time constant $\tau_{3}$ assigned to vibrational cooling is by a factor of two larger compared to the situation in methanol. The more rapid vibrational cooling in methanol compared to acetonitrile was reported for other systems and is related to intermolecular hydrogen-bonding in protic environments aggravating energy transfer from the solute to the solvent (Middleton et al., 2007; Ghosh et al., 2019).

There is an alternative rationale for the dynamics on the ps time scale. From resonance Raman studies it was concluded that the initial photoinduced process is an excited-state intramolecular proton transfer (ESIPT) (Lewis and Sandorfy, 1983). Since the isomer red I that predominates in solution is a hydrogen-bonded quasi-aromatic heterocycle, there is a striking congruence with intramolecularly H-bonded ß-diketones which have been studied with laser flash photolysis (Veierov et al., 1977; Kobayashi et al., 2013) and ultrafast spectroscopy approaches (Xu et al., 2004; Poisson et al., 2008; Verma et al., 2014; Verma et al., 2015; Verma et al., 2016). The latter revealed that ESIPT occurs within less than 0.1 ps, followed by relaxation to the lowest excited state in a few ps, and subsequent depopulation of the excited state on a time scale of 10 ps both by relaxation to the hydrogen-bonded ground-state isomer and the formation of other isomers by rotation around a single or double bond. Given the similarity of the molecular system and of the detected time scales, an analogous assignment of processes to the observed dynamics is plausible as well.

A quantitative analysis of our data assuming a fully branched model, in which each transient species is partially converting into each other and partially converting back into the ground-state species, gave a value of 7% for the quantum yield of product formation [see (Kutta et al., 2013), also containing detailed discussion on the general analysis of global fit data]. This is significantly lower than both isomer formation after ESIPT in ß-ketones [e.g., 36% for acetylacetone in acetonitrile (Verma et al., 2014)] and N=N isomerization in *trans*-azobenzene [31% for *n* **−** *π∗* and 15% for *π − π∗* excitation in acetonitrile, slightly different yields in other solvents (Quick et al., 2014)], so that an identification of the initial reaction step is not unambiguous from the TA data but will be further analyzed in Section 4. The rather low quantum yield could be related to the significant *π − π∗* character (delocalized over the entire molecular framework) of the initially excited S_2_ in TPF (see Supplementary Figure S6), but might also originate from the intramolecular hydrogen bond stabilizing the planar configuration of the six-membered chelated ring, thereby disfavoring an out-of-plane motion required for photoisomerization. Nonetheless, our TA data corroborates that although the vast majority of excited red I molecules returns to the red I configuration, the decision along which reaction pathway the system evolves involving several thermal isomerizations (vide infra) is already made within the first few ps.

### 3.2 ns-ms transient absorption of 1,3,5-triphenylformazan

**FIGURE 4:**
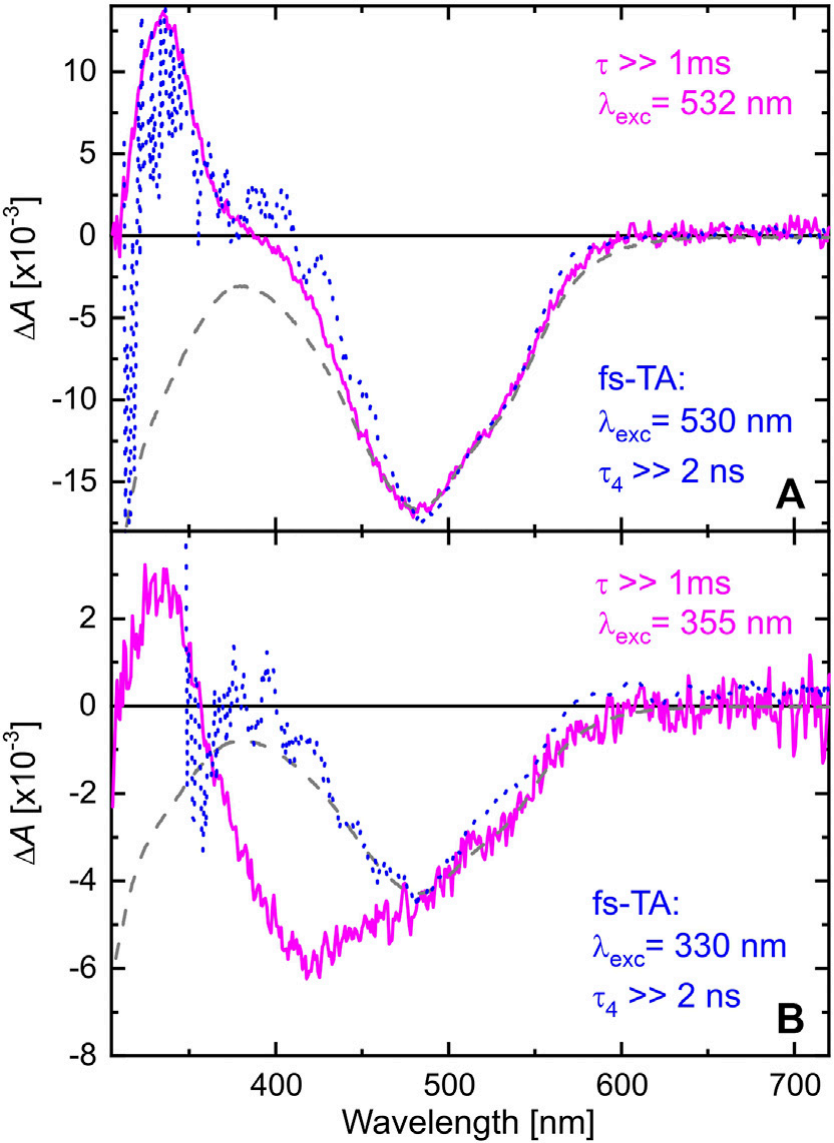
DADS of TPF in methanol after excitation at 532 nm **(A)** and 355 nm **(B)**. The magenta DADS result from a global monoexponential fit to the data on a 1 ms time window. The blue dashed curves are the DADS corresponding to $\tau_{4}$ of the fs-ns TA experiments (blue curves in Figures 3B,D) scaled to match the negative contribution at around 500 nm. The inverted absorption spectrum of the initial sample also scaled to the negative contribution at around 500 nm is given by a gray-dashed line for comparison.

In order to follow the reaction dynamics further on a ns to ms time scale, the TA of TPF after exciting at 532 or 355 nm was recorded with a pump-flashlamp-probe spectrometer using a streak camera as detection unit. The TA data recorded for TPF in methanol after excitation at 532 nm consist of one absorption band around 340 nm and the GSB at 480 nm. Both features rise faster than the temporal resolution of the used setup, which agrees with the formation of these two TA features within a few ps as determined in Section 3.1, and persist beyond the time window of 1 ms. Hence, a global monoexponential model yielding one DADS (magenta curve in Figure 4A) is sufficient to fully describe the data, and this DADS matches the one for $\tau_{4}$ of the fs-ns TA measurements (blue-dotted curve in Figure 4A).

When exciting at 355 nm, the same absorption band at 340 nm is observed as after 532 nm excitation. However, the GSB is broader in this case, ranging from 350 nm up to 600 nm. Comparing the DADS with the absorption spectrum of TPF under 520 nm illumination (black-dashed line in Figure 2) reveals the origin for this behavior. The intense Xe-flashlamp of ms duration used as probe source in the ns-ms TA experiment is sufficient to prepare a mixture of red I and yellow I isomers prior to the excitation of the system by the intense pump pulse so that exciting at 532 nm leaves the yellow isomers unaffected, whereas exciting at 355 nm allows the excitation of both isomers, causing a GSB signal comprising the spectral signature of both. To note, in the fs-ns TA experiment (blue-dashed line in Figure 4B) no accumulation of a probe-light induced photostationary equilibrium between forms red I and yellow I is observed due to significantly shorter and less intense probe pulses.

Again, the ns-ms TA data obtained for TPF in acetonitrile (Supplementary Figure S2) are very similar to the ones recorded in methanol.

### 3.3 ms–min transient absorption of 1,3,5-triphenylformazan

**FIGURE 5:**
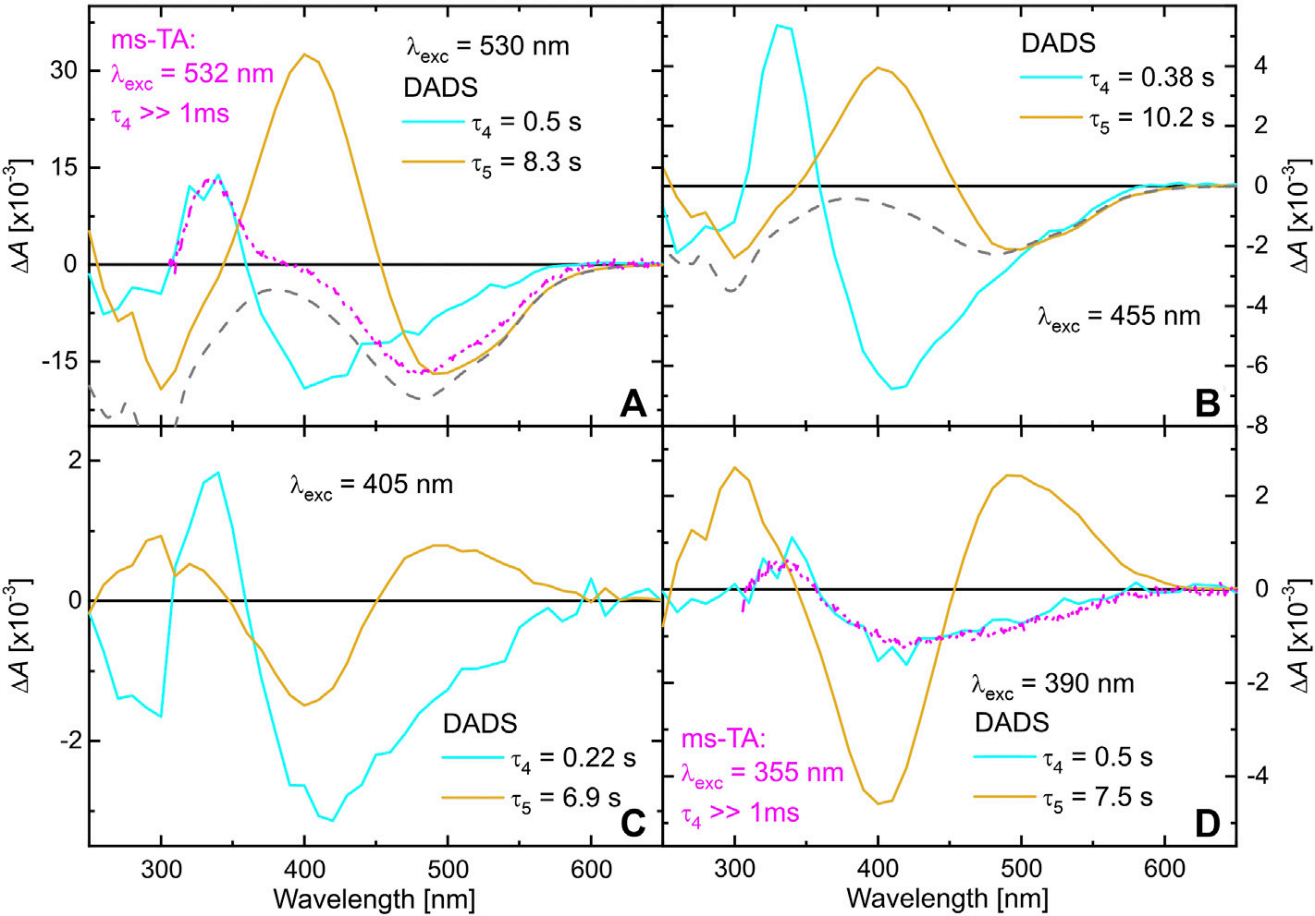
DADS of TPF dissolved in methanol, obtained by a global biexponential fit to the data matrices detected after excitation at 530 nm **(A)**, 455 nm **(B)**, 405 nm **(C)** and 390 nm **(D)**. The gray-dashed line represents the absorption spectrum of the solution, while the pink DADS are taken from Figure 4 for comparison.

The preceding two TA measurement series have shown that the species absorbing around 340 nm is formed on an ultrafast time scale and persists well beyond 1 ms. Hence, ms to min TA measurements were performed exciting TPF either at 390, 405, 455, or 530 nm. The maximal time window of all four measurements was set to 1 min, sufficient for detecting the full recovering process to the initial ground state situation of TPF in methanol. All data matrices were analyzed by a global biexponential fit, yielding the DADS shown in Figure 5.

The cyan DADS corresponding to $\tau_{4}\;$ exhibits a positive absorption peaking around 340 nm for all excitation wavelengths. Comparison to the DADS from the ns-ms TA measurements (pink curve in Figures 5A,D) confirms that the same dynamics that were monitored up to 1 ms can now be followed completely. This absorption band was already identified in toluene solution (Wortmann et al., 2022) and assigned to the red II form of TPF, in accordance with the isomerization scheme of Figure 1. The DADS furthermore comprises negative contributions around 500 nm and around 405 nm. These negative amplitudes resemble spectral features of red I and yellow I, respectively, and thus give evidence that photochemically formed red II thermally relaxes back to red I and to yellow I with a decay time of a few hundred milliseconds. The typical absorption feature of formed yellow I is seen at 405 nm in the orange DADS corresponding to $\tau_{5}$ . Thus, the slowest dynamics observed after 530 nm excitation are described by the orange DADS, which shows the spectral features of red I as a negative amplitude (compare the gray-dashed inverted ground-state absorption spectrum) and the absorption feature of yellow I as a positive amplitude, demonstrating the thermally activated back relaxation to red I on a time scale of 10 s (Figure 5A).

Figure 5B shows a similar experiment, but with 455 nm excitation. As red I and red II both absorb at 455 nm, here contributions of both isomers show up in the data. The observed dynamics can nonetheless be interpreted on the lines of the 530 nm excitation experiment in Figure 5A, as the additional contribution of excited red II are rather low.

The situation changes when exciting TPF at 405 nm (Figure 5C). Now, only a small amount of red I is excited, again giving rise to the dynamics described by the cyan DADS that is very similar to the two situations with 530 or 455 nm excitation, respectively. However, since in the dark also yellow I contributes to a small amount to the ground state equilibrium (see Figure 1) it is excited by 405 nm light giving rise to yellow II formation. This is substantiated by the second DADS (orange) that shows a positive absorption feature of yellow II at around 500 nm that decays with a lifetime of ca. 7 s (Figure 5C) back into yellow I identified by the characteristic negative amplitude at 405 nm. The data recorded with 390 nm excitation (Figure 5D) agrees with this interpretation, because at this wavelength, red I can be excited even worse, whereas yellow I still has a high extinction coefficient, so that the cyan DADS (mostly representing relaxation after generation of red II from red I) becomes smaller whereas the orange DADS (comprising yellow II photogenerated from yellow I) gains in relative intensity.

In comparison to the kinetics in methanol, the ground-state processes of TPF on a ms to min time scale are slower in acetonitrile (see Supplementary Figure S3), but much faster than in toluene solution (Wortmann et al., 2022). There, it was shown that the solvent’s ability to participate in hydrogen bonds is decisive for how fast the equilibration proceeds.

In the following, we try to develop a line of reasoning with the help of quantum-chemical calculations for how the isomers are interconnected, explaining the slow processes observed for TPF after photoexcitation.

## 4 Analysis of reaction pathways

### 4.1 Simulation of ground-state conformers

Most of the previous experimental studies on the photochemical processes of TPF do not differentiate possible rotamers beyond the four isomers obtained from the cis-trans isomerizations around the N=N and the C=N double bond, resulting in the trans-syn nomenclature also displayed in Figure 1. Theoretical studies [e.g., (Buemi et al., 1998; King and Murrin, 2004)] have addressed further rotamers, as we will also do in the following. For our discussion of the reaction pathways, we further take into account the rotations around the two adjacent single bonds, so that each of the four isomers labelled in Figure 1 may lead to four distinguishable conformers. Our calculations at the B3LYP/def2-TZVP/D4 level of theory indeed result in 16 corresponding ground-state minima, i.e., 16 potentially stable conformers, which however strongly differ in energy and the height of the barriers connecting them.

**FIGURE 6:**
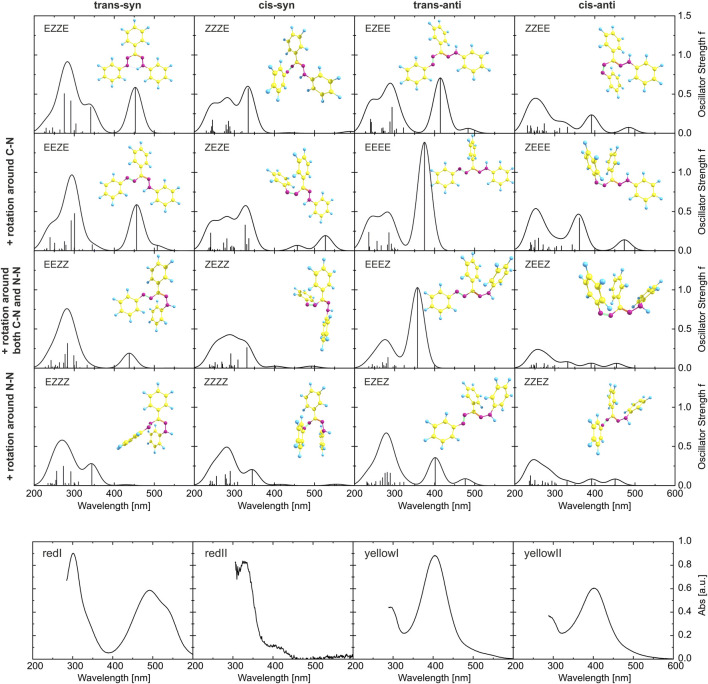
Top 16 panels: Results from DFT calculations for the 16 isomers considered in this study. The optimized ground-state geometries are displayed together with the calculated absorption spectra (the oscillator strengths at the corresponding transition energies are given as stick spectrum convoluted each with a Gaussian of a 40 nm width at half maximum). The orientations with respect to the N=N and C=N double bonds are identical within each column, whereas the ones with respect to the C–N and N–N single bond are the same for each row. Bottom 4 panels: Experimentally determined spectra of the four isomers corresponding to the double-bond orientations given at the top of each column. The red I spectrum is the ground-state absorption spectrum of a toluene solution, yellow I was measured directly after illumination with 455 nm of a toluene solution, yellow II corresponds to the photostationary state in toluene induced by 455 nm light (Wortmann et al., 2022), and red II is estimated by subtracting the GSB from the pink DADS in Figure 4A.

The optimized structures of the 16 conformers are plotted in Figure 6, together with the absorption spectra obtained from the calculations. For a systematic description, we use the E/Z nomenclature and start from the N=N double bond. Thus, the most stable isomer (red I, trans-syn) with the intramolecular hydrogen bond is the EZZE conformer, reflecting the E (trans) configuration with regard to the N=N double bond, Z with regard to N–C, Z (syn) for C=N, and E for the N–N single bond, respectively. Each row of panels in Figure 6 represents a variation of a single bond, as indicated on the left, whereas each column represents an isomerization of a double bond.

The absorption spectra, even within one column and hence only due to rotation around single bonds, vary substantially (for a comparison with calculations using the CAM-B3LYP functional and including a conductor-like polarizable continuum model, see Supplementary Figures S4, S5). To allow a comparison to experiment, the lowest row of panels in Figure 6 gives experimental absorption spectra, where always one isomeric species is dominating. Starting on the left with the absorption spectrum of the solution in the dark (corresponding to red I), followed by the spectrum of red II derived from spectra of intermediates in time-resolved experiments, additionally to the spectrum of yellow I measured directly after illumination and the spectrum of yellow II obtained in the photostationary state. In the visible spectral range, the simulated spectra of EZZE and EEZE are very similar and match best with the experimental spectrum corresponding to red I, although the experimental data is red-shifted and exhibits a shoulder at longer wavelengths not reproduced in the simulations. Both aspects might be related to a significant stabilization effect caused by the intramolecular hydrogen bond, which the calculations might underestimate. The spectrum of the intermediate red II matches best with ZZZE or ZEZE, substantiating that the initial process after photoexcitation involves a trans-cis isomerization around the N=N double bond. Comparing the experimental and theoretical spectra, also the assignment of EZEE as the yellow I species is confirmed. Furthermore, a comparison of the experimental spectrum of yellow II with the calculated spectra in the right-hand column yields the best agreement for the ZZEE conformer, whose absorption peaks around 400 and 490 nm might not be identifiable as separate peaks in the experimental spectrum.

While the juxtaposition of the theoretical with the experimental spectra from time-resolved measurements confirms the involvement of the four species initially included in the Kuhn-Weitz and Grummt-Langbein reaction schemes (see Figure 1), the actual sequence of transformations cannot be deduced. Especially, the thermal process from red II (ZEZE) to yellow I (EZEE) in the Grummt-Langbein scheme (diagonal arrow in Figure 1) formally necessitates isomerization around both the N=N and the C=N double bond. Experiments indicated that this might be a bimolecular process and that two TPF molecules are required (Grummt and Langbein, 1981). In the following section, we provide an analysis for identifying which conformers are involved.

### 4.2 Interconnection among isomers

**FIGURE 7:**
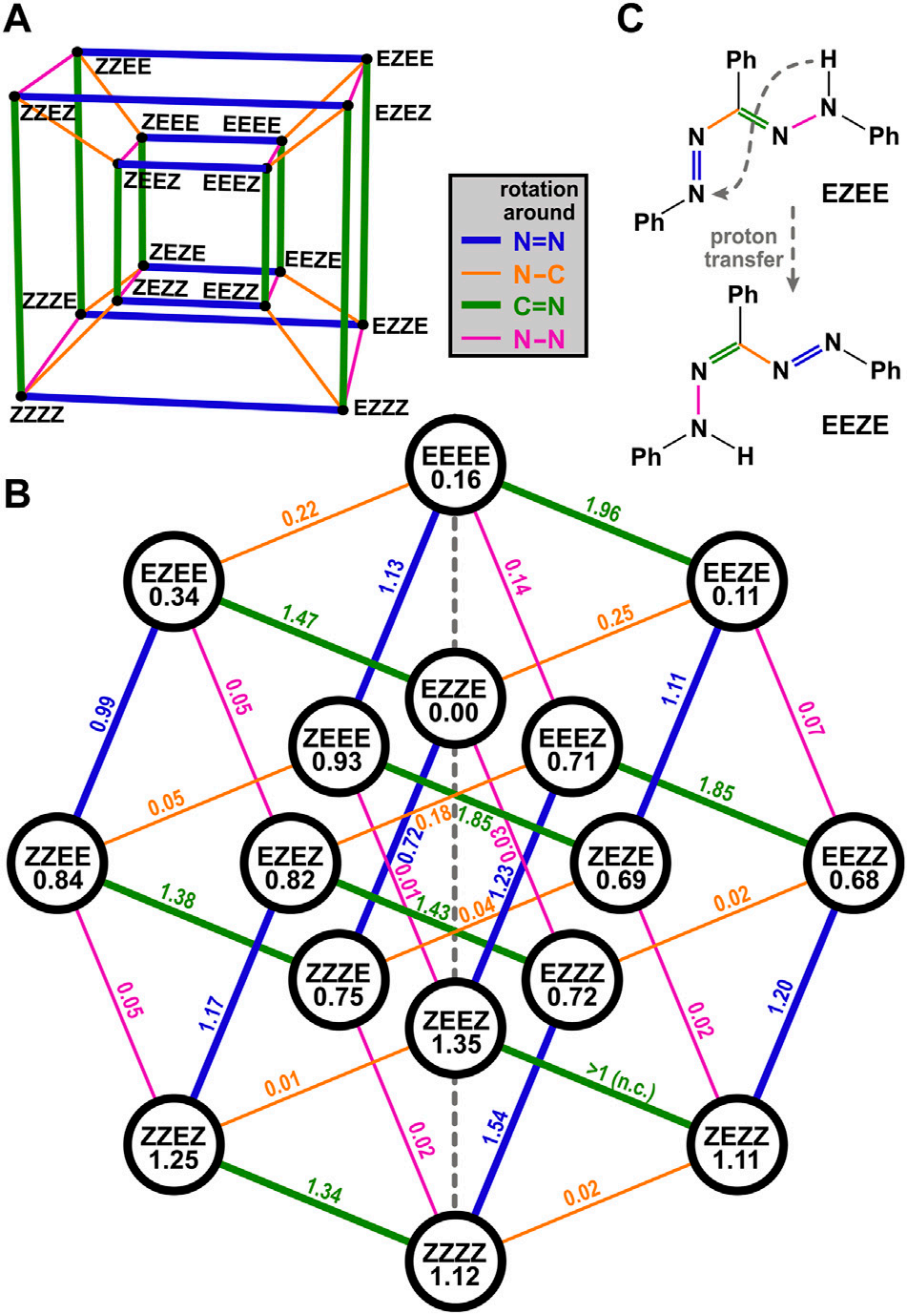
Reaction pathways illustrated as Schlegel diagram **(A)** or hypercube graph **(B)**. The isomers’ calculated energies, referenced to the energetically most stable geometry EZZE, are written in the circles, barrier heights are shown above the reaction pathway; all values are in eV. The final single point energies of the individual isomers were optimized by DFT calculations on the B3LYP/def2-TZVP level of theory. The energy barriers between two adjacent isomeric species of TPF are roughly estimated by relaxed surface scans along a fixed torsional angle. The stated values along the possible pathways always describe the amount of energy to overcome the energy barrier from the energetically higher lying isomer to the energetically more favored species. The performed calculations are summarized in Supplementary Figure S8. Note that in case of one barrier height the calculation did not converge (n.c.). **(C)** exemplarily displays that a proton transfer from the phenylhydrazone to the azo group is associated with the formation of an isomer with reversed nomenclature.

For merocyanine systems in which eight cis/trans isomers can occur, it is illustrative to represent them as the corners of a cube, so that each edge of the cube corresponds to a change of one orientation from cis to trans or vice versa (Ernsting et al., 1990). For an analogous treatment of TPF and the 16 structures shown in Figure 6, a four-dimensional hypercube, often called tesseract, would be required, which has 16 corners (i.e., isomers) and 32 edges (i.e., reaction pathways involving one rotation). In order to plot it in two dimensions, the tesseract can be represented for instance by its Schlegel diagram (a perspective 3D view, with the fourth dimension pointing inwards, Figure 7A) or by an orthogonal projection (Coxeter, 1948), from which the hypercube graph Q_4_ is obtained (Maehara, 2016; Hammack and Kainen, 2021). Transferred to the TPF isomer manifold (Figure 7B), each of the 16 isomers is thus connected to four other isomers by a line. As follows from the properties of hypercube graphs (Hammack and Kainen, 2021), the lines can be separated into 4 groups, which correspond to the 4 possible rotational degrees of freedom in TPF as indicated by the color in Figure 7B. Note that each isomer is connected to one line of each color (as there are four possible rotations), and lines of identical color are parallel in this representation.

We have further calculated the energy of the 16 isomers (given in the circles together with the abbreviation of the isomer) as well as the 32 barriers (values given above the lines representing the rotation) to go from one isomer to another. In this way, one can visualize nicely how the reaction might proceed.

The EZZE (red I) isomer is the energetically most favorable one, and all four possible rotations to reach another isomer are energetically uphill, exhibit a significant barrier, or both. As inferred from the experiments, EZZE performs a trans/cis isomerization around the N=N bond (i.e., it follows the blue line in Figure 7B) upon photoexcitation, reaching ZZZE. Looking at the possible pathways of ZZZE, one can deduce that an almost barrierless and slightly downhill pathway is possible by rotation around the N–C bond (orange line), yielding ZEZE (isomer red II). From there, no further rotation is plausible, because of too high barriers and energetically disfavored isomers.

So how is it possible that a thermal process leads from ZEZE (red II) to EZEE (yellow I) without any detectable intermediates? In chelated formazan isomers exhibiting an intramolecular hydrogen bond, intramolecular proton transfer was reported to occur in the ground state (Fischer et al., 1968; Hutton and Irving, 1980; Hutton and Irving, 1982; Grummt et al., 1984), and also IR spectra in solution and in the solid state point towards this pathway (Otting and Neugebauer, 1968; Otting and Neugebauer, 1969). A combined IR and resonance Raman study, also of unsymmetrical derivates of TPF, found evidence for this tautomerism even in the photochemical generation of non-chelated isomers and interpreted the transfer step to occur in the excited state (Lewis and Sandorfy, 1983). Owing to the symmetry of the TPF molecule, transferring a proton from the phenylhydrazone to the azo group reverts the order of single and double bonds. Thus, the nomenclature is reverted, as exemplarily shown in Figure 7C. In the hypercube graph of Figure 7B, this means that such a proton transfer eventually is equivalent to a reflection on the central vertical line (dashed in gray).

Hence, for chelated isomer ZEZE (red II), the next step might be a proton transfer, resulting in the formation of EZEZ, which will immediately relax to the energetically lower-lying EZEE (yellow I). Therefore, we infer that for the thermal process from red II to yellow I in Figure 1, it is not necessary to isomerize around both double bonds, but only to transfer a proton.

The same rationale can also explain how the reaction proceeds further. From EZEE (yellow I), a return to the most stable isomer EZZE (red I) formally necessitates a thermal rotation around the C=N double bond. If the proton is transferred, EEZE is obtained, from where it is much easier to reach EZZE because only a rotation around the N–C single bond is required.

While for chelated isomers, the proton transfer may proceed directly, for other isomers the distance between the donating and the accepting nitrogen atom is too far. In principle, also an intermediate [as is of relevance in formazan synthesis (Hegarty and Scott, 1966; Hegarty and Scott, 1967; King and Murrin, 2004)] with two azo groups and the H atom at the interjacent C atom is conceivable, but much less stable than the formazan tautomers (Buemi et al., 1998) (see also Supplementary Figure S7). However, the transfer might occur *via* a proton wire mechanism, i.e., in a Grotthuss-type fashion (Agmon, 1995; Miyake and Rolandi, 2015; Adams et al., 2021) as was found in water but also identified in other protic solvents (Stoyanov et al., 2008; Fujii et al., 2018; Long et al., 2020). Hence, the isomerization involving proton transfer should only be possible in protic solvents or in aprotic solvents containing at least traces of protic cosolvents. Indeed, the thermal isomerization from EZEE (yellow I) to EZZE (red I) occurs extremely slow in thoroughly dried toluene, and values up to 138.9 h (Kuhn and Weitz, 1953) are reported for the half-life of EZEE. Addition of slight amounts of protic solvents drastically accelerate the reaction (Kuhn and Weitz, 1953; Sueishi and Nishimura, 1983), and in case of alcohols as a cosolvent, a correlation with the H-bonding donating ability of the alcohol was found (Wortmann et al., 2022). Hence, while hydrogen bonding to nitrogen atoms being part of a double bond may also facilitate a ground-state isomerization, in the case of TPF an actual proton transfer might contribute to a significant extent.

## 5 Summary and conclusion

The primary reaction step when exciting TPF with light is an isomerization around the N=N double bond through which the electronic ground state is reached on an ultrafast time scale. Our combined experimental and theoretical study unveiled that the initially excited conformer EZZE (red I) thus turns into ZZZE and from there directly to ZEZE. The low barrier found for the latter step might even imply that the excited-state isomerization and rotation around the N–C single bond might proceed in a concerted fashion. Following spectroscopically the evolution of the newly formed ZEZE (red II), it was shown that the next reaction step occurs on a time scale of hundreds of milliseconds, yielding EZEE (yellow I). For this reaction step, we propose that an intramolecular proton transfer significantly contributes, so that no isomerization around a double bond is necessary. Along these lines, the reaction step from EZEE back to EZZE might also include a proton transfer and proceed *via* EEZE, so that again no rotation around a double bond is required.

The above conclusion are supported by observations in different solvents and interpreted in the context of a Grotthuss-type mechanism, also explaining the remarkably high decay times reported for the last step of the TPF photocycle in dried aprotic solvents. It might be worthwhile to investigate whether also in molecules with a larger separation between the azo and the hydrazone group, a similar acceleration of the ground-state equilibration is found in protic solvent environments. These aspects could also be of interest in the field of organocatalysis, where hydrazone compounds find increased attention (Müller and List, 2009; Landge et al., 2011; Müller et al., 2011; de Gracia Retamosa et al., 2016; Aprahamian, 2017; Cvrtila et al., 2017; Mader et al., 2022; Žabka and Gschwind, 2022).

## Data Availability

The raw data supporting the conclusion of this article will be made available by the authors, without undue reservation.
