# Supplementary material for: Monitoring the photochemistry of a formazan over 15 orders of magnitude in time
Source: Front Chem. 2022 Sep 28;10:983342. doi: 10.3389/fchem.2022.983342 (PMC9554553; doi:10.3389/fchem.2022.983342)
Supplement: Supplementary file 1 [file DataSheet1.PDF]

Supplementary Material

1 fs-ns TA of TPF

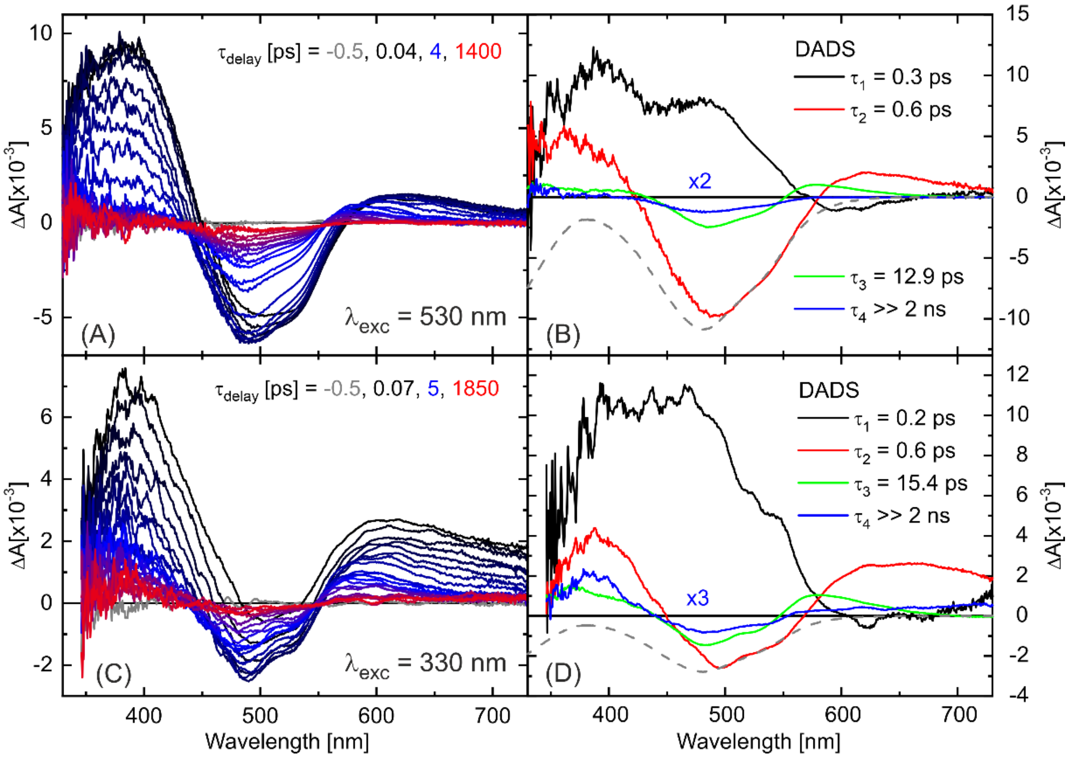

Supplementary Figure 1. Transient absorption of TPF in acetonitrile after excitation at 530 nm (A) and 330 nm (C) after defined delay times on a fs-ns time scale. The corresponding DADS from a global exponential fit to the data are given in B and D. The scaled and inverted absorption spectrum of the initial sample is given by a gray dashed line for comparison.

2 ns-ms TA of TPF

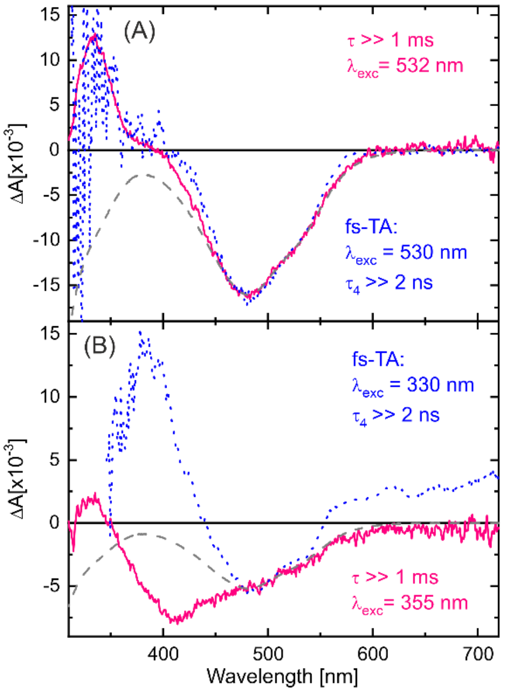

Supplementary Figure 2. DADS of TPF in acetonitrile after excitation at 532 nm (A) and 355 nm (B). The magenta DADS result from a global monoexponential fit to the data on a 1 ms time window. The blue dashed curves are the DADS corresponding to τ_4 of the fs-ns TA experiments (blue curves in Supplementary Figure 1B/D) scaled to match the negative contribution at around 500 nm. The inverted absorption spectrum of the initial sample also scaled to the negative contribution around 500 nm is given by a gray dashed line for comparison.

3 ms-min TA of TPF

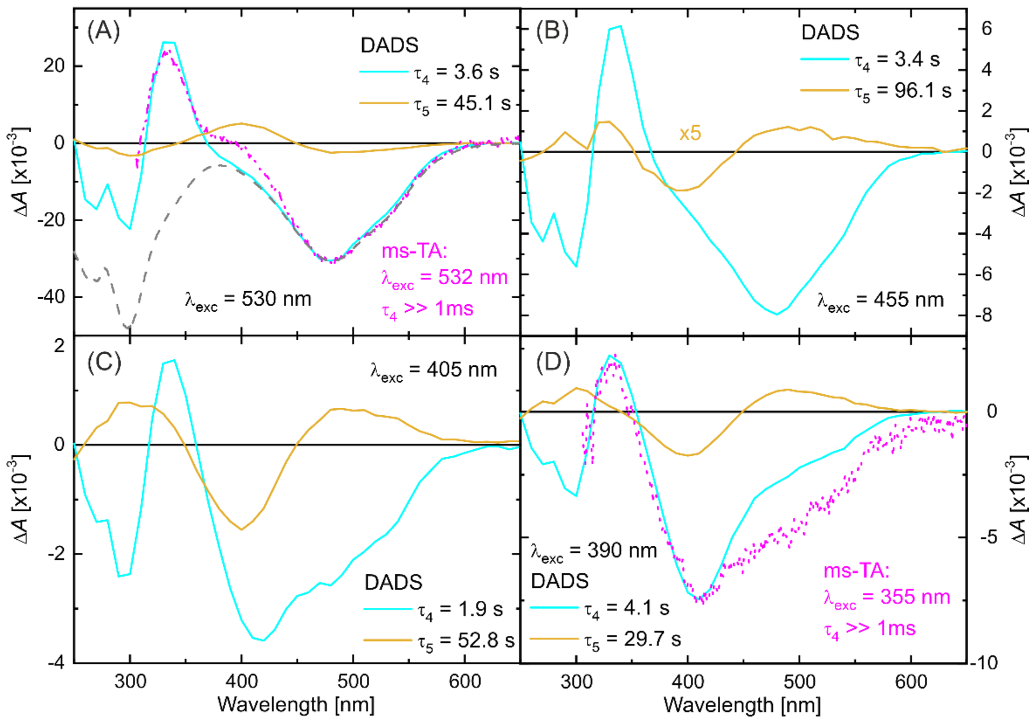

Supplementary Figure 3. DADS of TPF dissolved in acetonitrile obtained by a global biexponential fit to the data matrices detected after excitation at 530 nm (A), 455 nm (B), 405 nm (C) and 390 nm (D). The gray dashed line represents the inverse absorption spectrum of the initial solution, while the pink DADS are taken from Supplementary Figure 2 for comparison.

4 Quantum-chemical Calculations

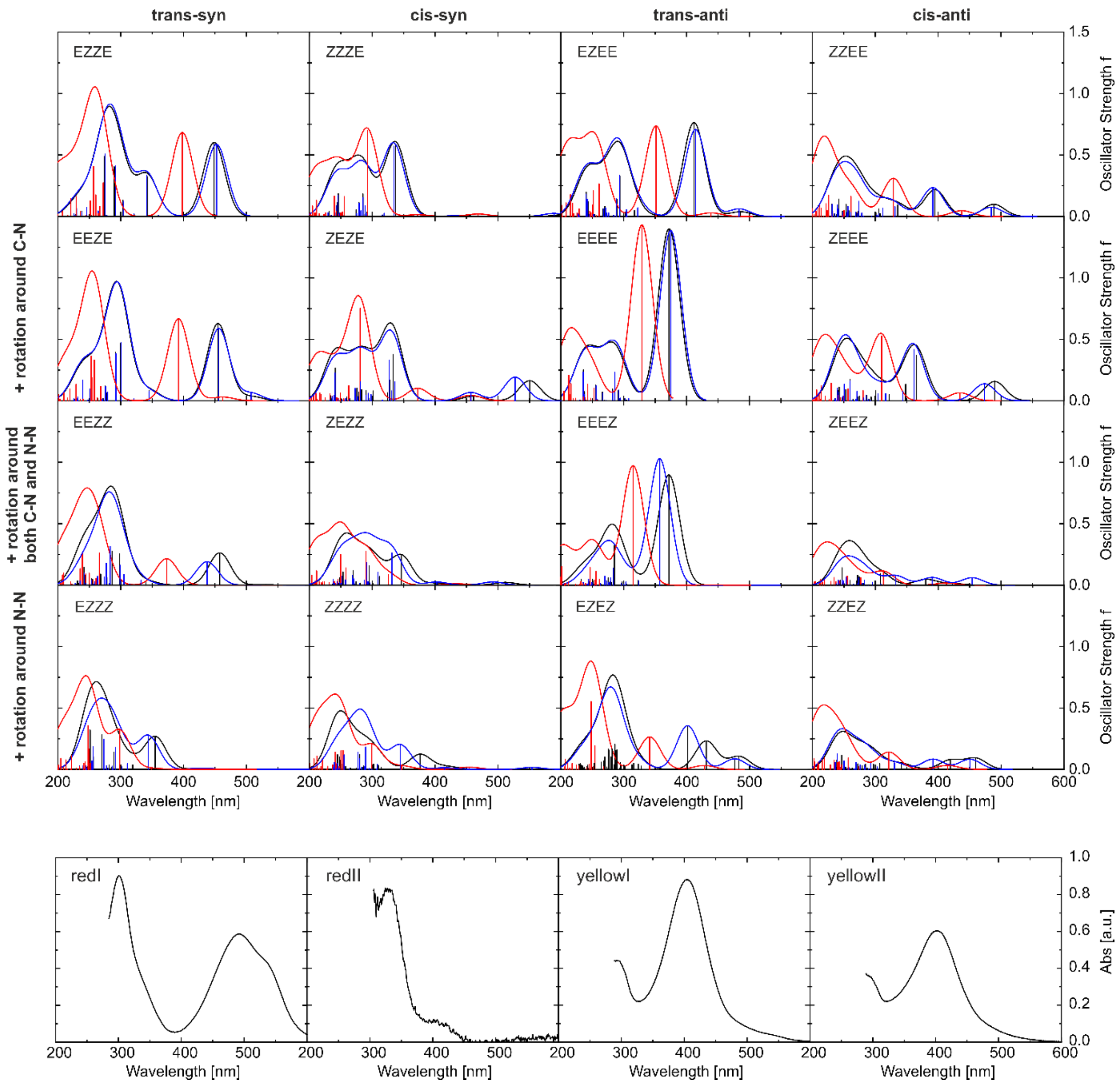

Supplementary Figure 4. Comparison of the calculated absorption spectra using B3LYP/def2-TZVP (black), B3LYP/def2-TZVP/D4 (blue) and CAM-B3LYP/def2-TZVP/D4 (red). The stick spectra were convoluted with Gaussians of 40 nm width at half maximum in all cases. The orientations with respect to the N=N and C=N double bonds are identical within each column, whereas the ones with respect to the C–N and N–N single bond are the same for each row. For the bottom row, refer to the description of the experimental spectra in the caption of Figure 6 of the main manuscript.

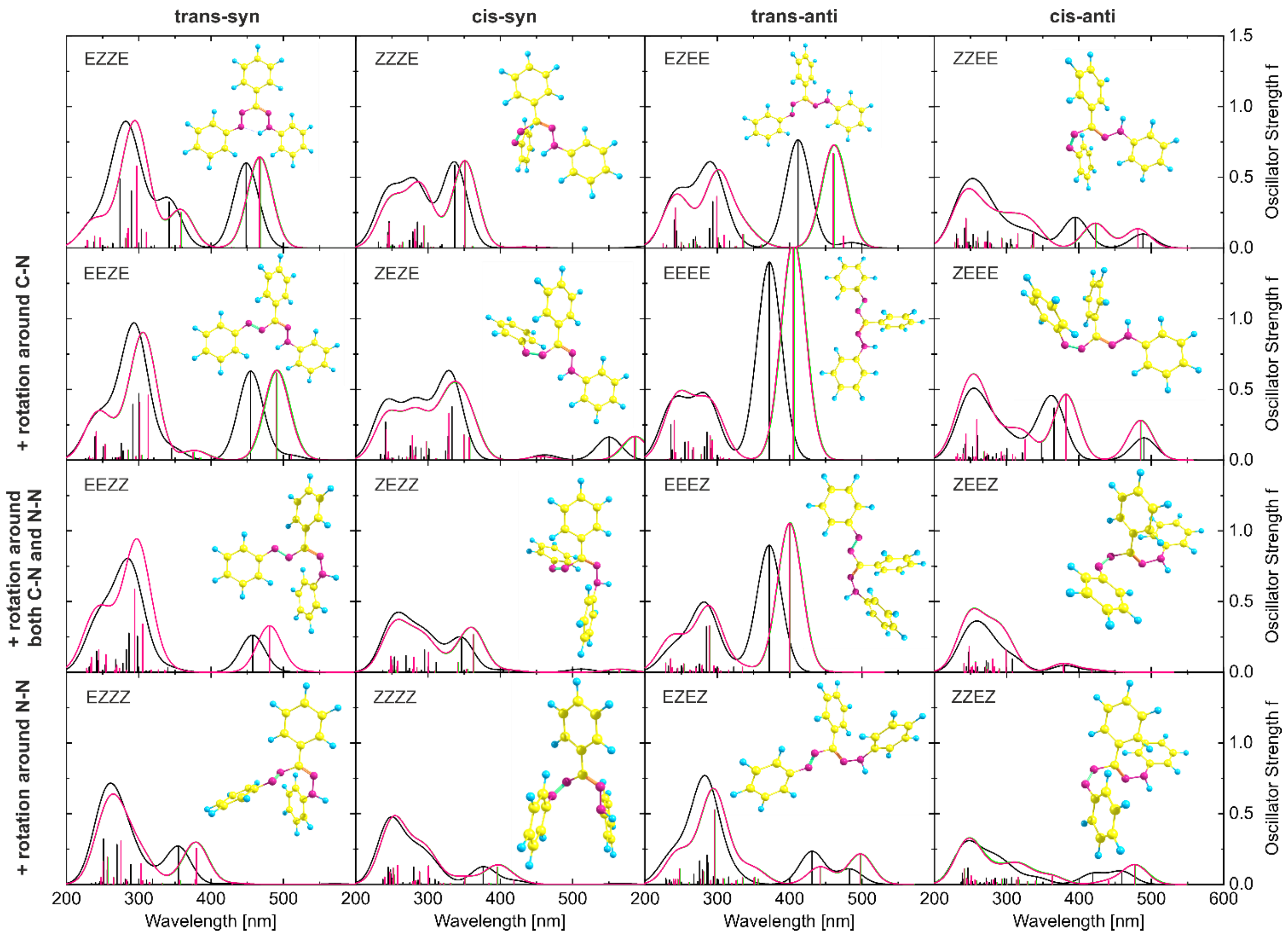

Supplementary Figure 5. Results from DFT (B3LYP/def2-TZVP; black = gas phase, magenta = CPCM(methanol); green = CPCM(acetonitrile)) calculations for the 16 isomers considered in this study. The optimized ground-state geometries are displayed together with the calculated absorption spectra (the oscillator strengths at the corresponding transition energies are given as stick spectrum convoluted each with a Gaussian of a 40 nm width at half maximum). The orientations with respect to the N=N and C=N double bonds are identical within each column, whereas the ones with respect to the C–N and N–N single bond are the same for each row.

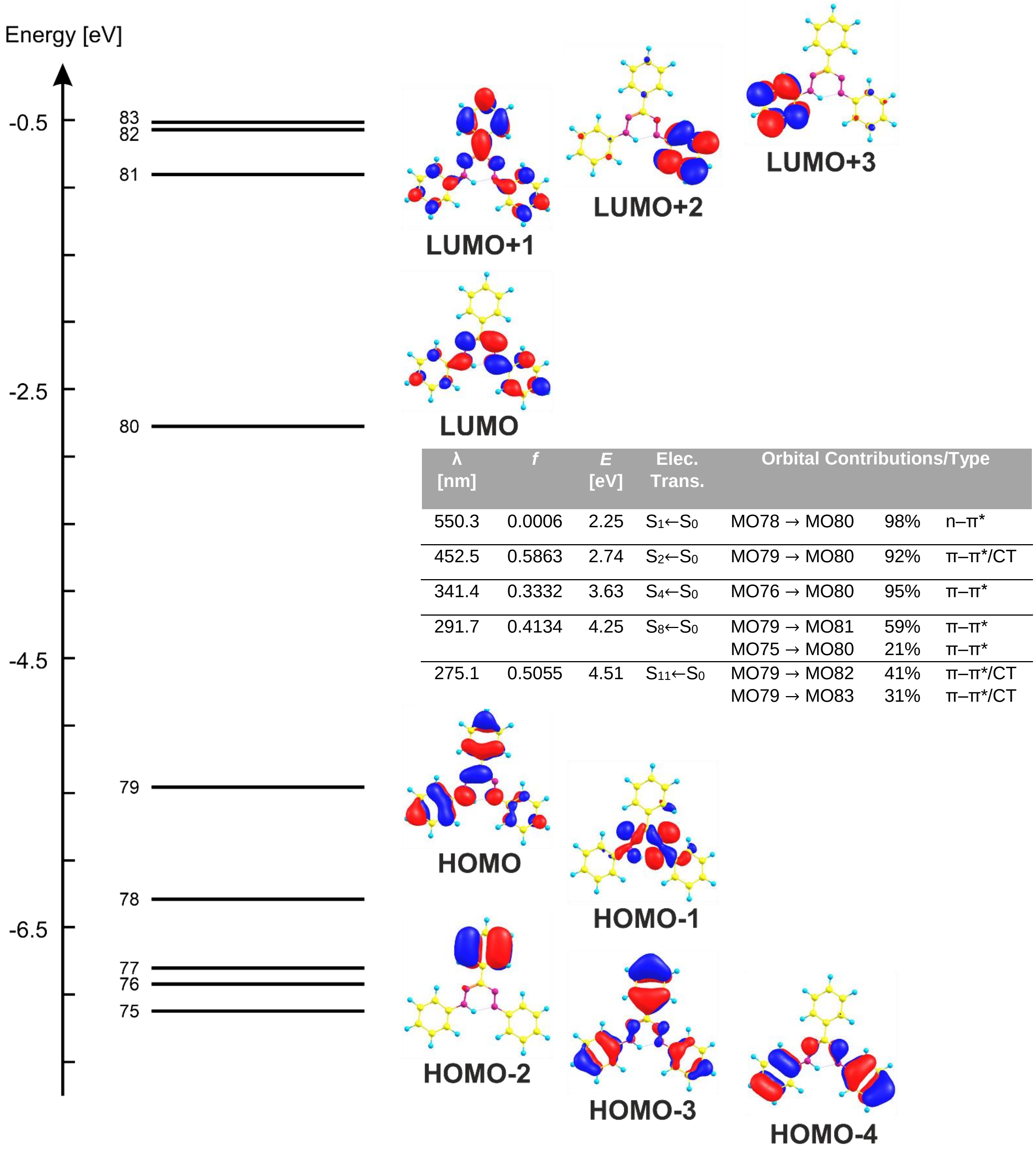

λ [nm]	*f*	*E* [eV]	Elec. Trans.	Orbital Contributions/Type		
550.3	0.0006	2.25	$S_1 \leftarrow S_0$	MO78 → MO80	98%	n–π*
452.5	0.5863	2.74	$S_2 \leftarrow S_0$	MO79 → MO80	92%	π–π*/CT
341.4	0.3332	3.63	$S_4 \leftarrow S_0$	MO76 → MO80	95%	π–π*
291.7	0.4134	4.25	$S_8 \leftarrow S_0$	MO79 → MO81<br>MO75 → MO80	59%<br>21%	π–π*<br>π–π*
275.1	0.5055	4.51	$S_{11} \leftarrow S_0$	MO79 → MO82<br>MO79 → MO83	41%<br>31%	π–π*/CT<br>π–π*/CT

Supplementary Figure 6. Molecular orbitals of the EZZE isomer based on TD-DFT calculations with B3LYP/def2-TZVP/D4 level of theory. The relevant highest occupied (HOMO) and lowest unoccupied molecular orbitals (LUMO) are represented as isosurface (contour value = 0.03). The table summarizes the calculated vertical transition energy, oscillator strength *f*, involved orbitals with contributions > 10%, and transition type of the four main transition bands of EZZE. Also the lowest electronic transition of n – π* character is included.

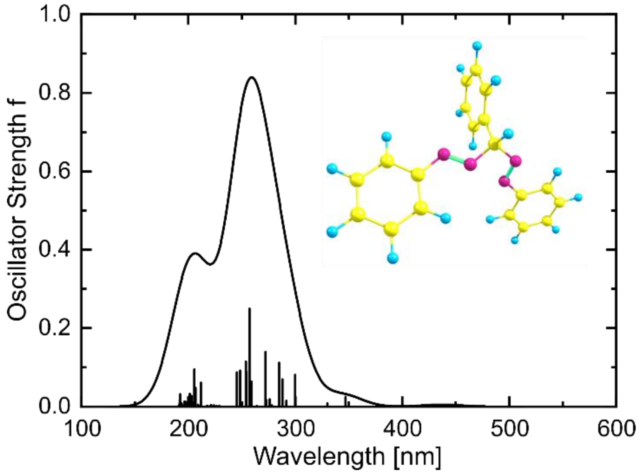

Supplementary Figure 7. Results from a DFT (B3LYP/def2-TZVP/D4) calculation for a structure where the hydrogen atom is bound to the carbon atom rather than to a nitrogen atom like in TPF, resulting in two azo groups instead of a combination of one azo and one hydrazone group as in TPF. The optimized ground-state geometry is displayed together with the calculated absorption spectrum (the oscillator strengths at the corresponding transition energies are given as stick spectrum convoluted with a Gaussian of a 40 nm width at half maximum).

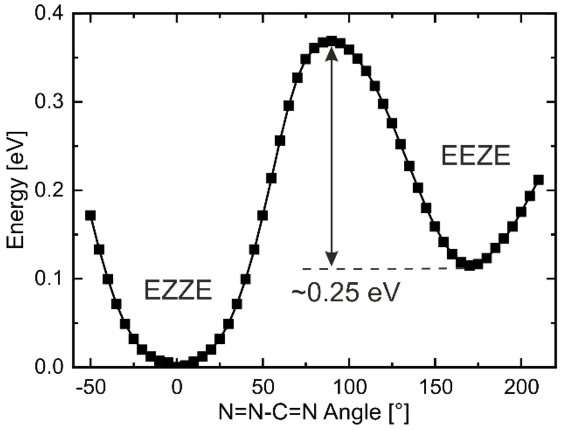

(A): rotation was monitored in 5° steps

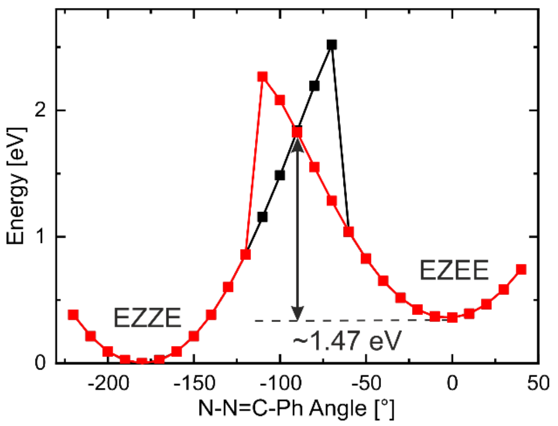

(B): Orca 4.2.1

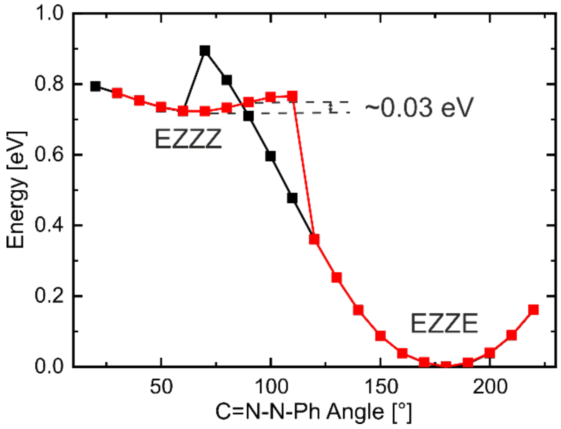

(C): -

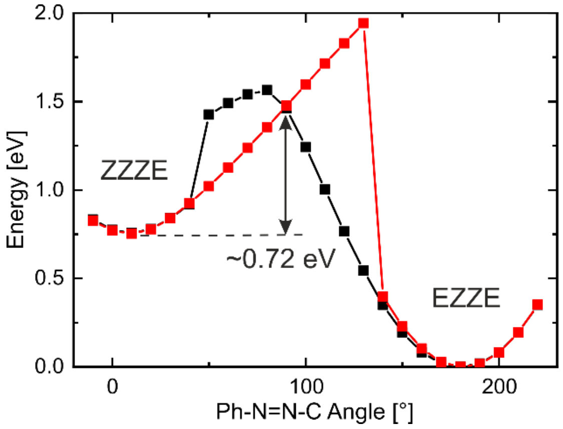

(D): Orca 4.2.1, Geom. Constraint: N-N=C 121.36° / BS: (Ph)C-N-N=C 173.921°

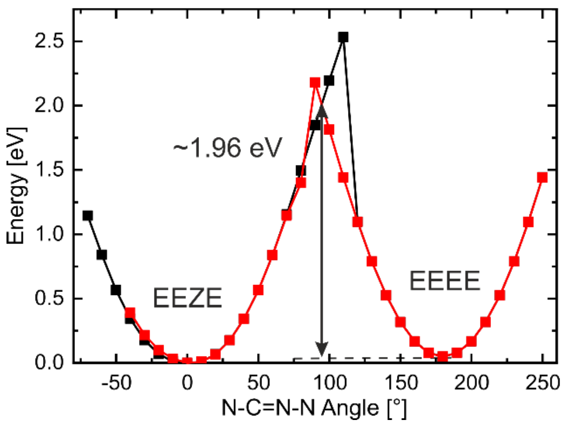

(E): -

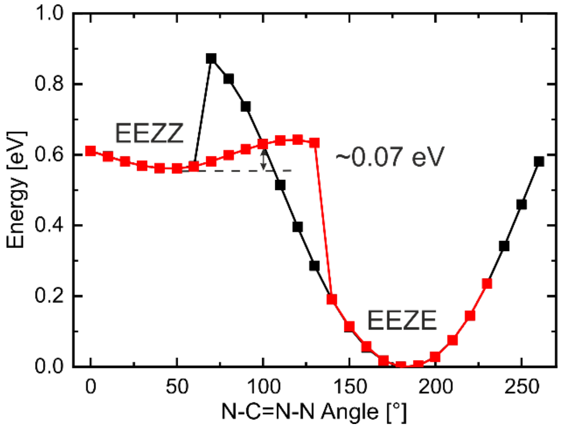

(F): -

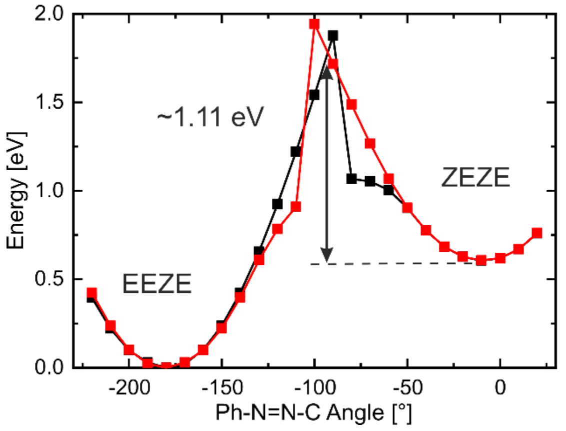

(G): Geom. Constraint: C-N=N 120° (both)

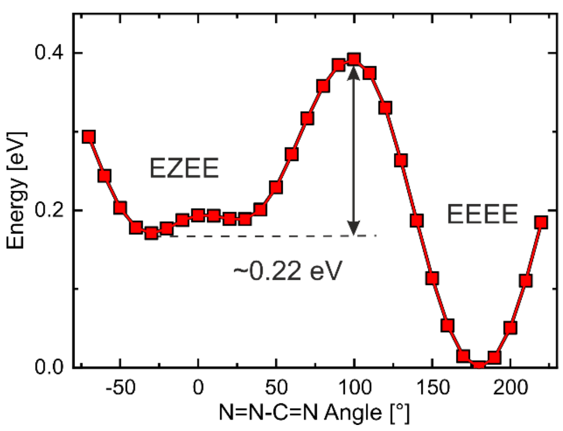

(H): -

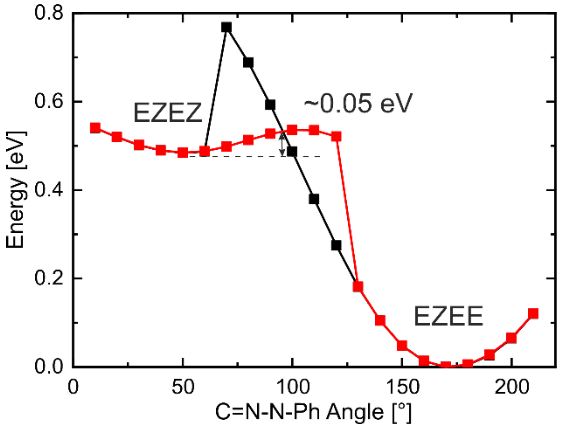

(I): -

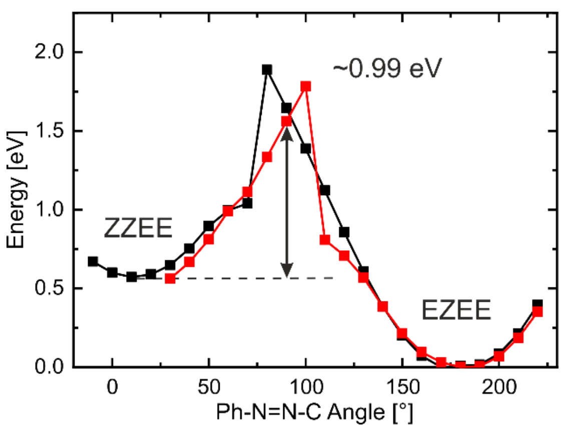

(J): Geom. Constraint BS: C-N=N 125.551°

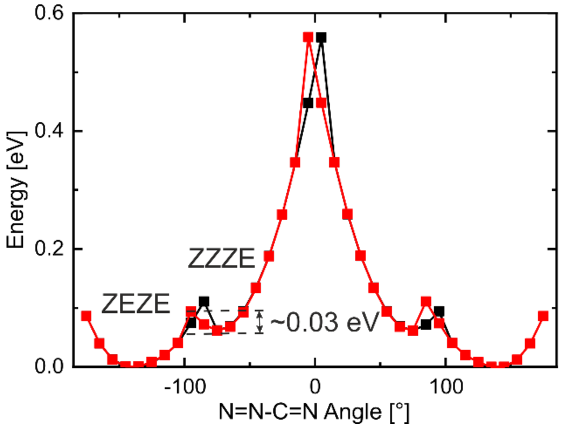

(K): -

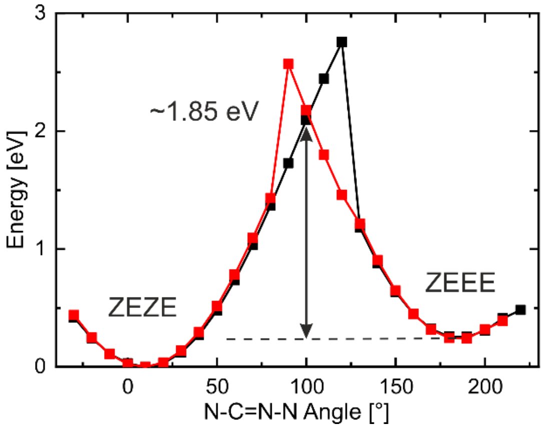

(L): Geom. Constraint: C-N=N-C(Ph) 12°(both), C=N-N 120°(both), C-N=N 124° (BS only), C-N=N-H 1° (BS only)

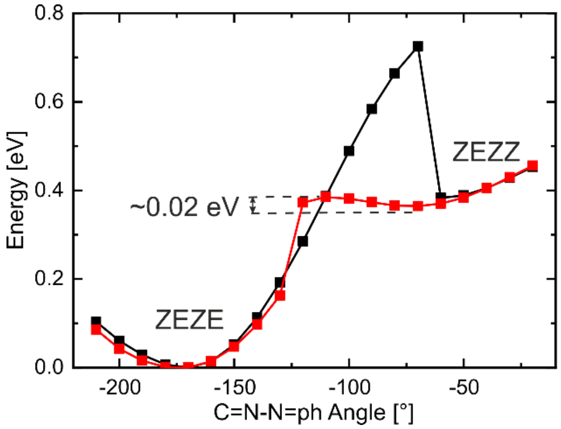

(M): Geom. Constraint: N=C-N=N 254°

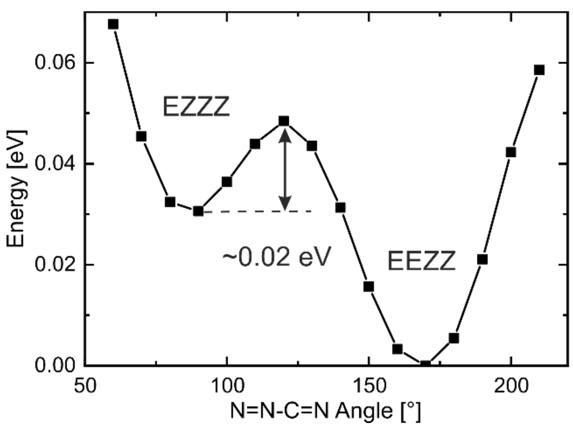

(N): -

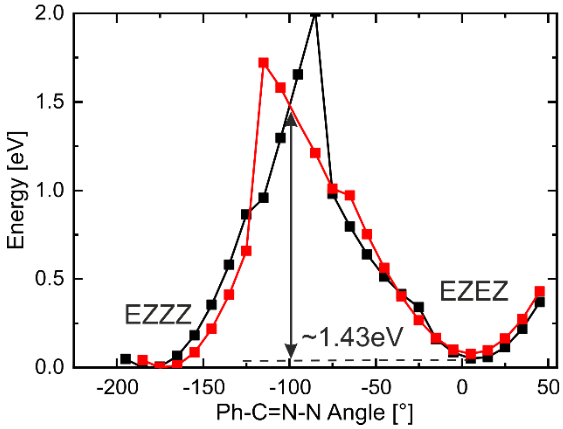

(O): Geom. Constraint: C=N-N 124°, C=N-N-C(Ph) 50°, C-N=N-C(Ph) 178° (both)

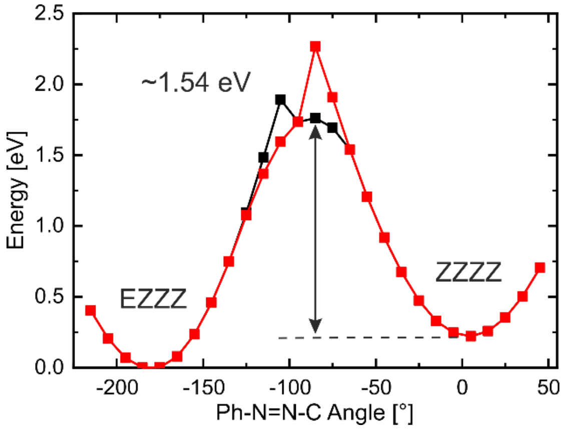

(P): Geom. Constraint: C=N-N-H 158°, C-N=N 125°, N=C-N=N 75°, N=N-C(Ph) 123° (both)

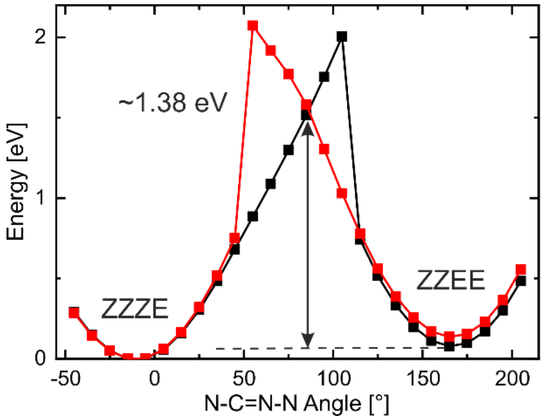

(Q): Geom. Constraint: C=N-N 121°, C-N=N 125° (both), C-N=N-C(Ph) 10°(both), N=N-C(Ph)-C(Ph) 30° (only BS)

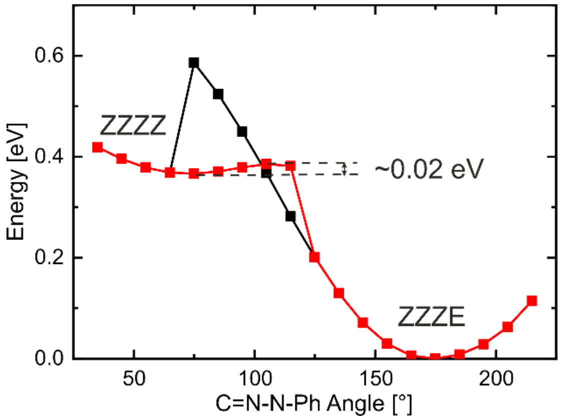

(R): -

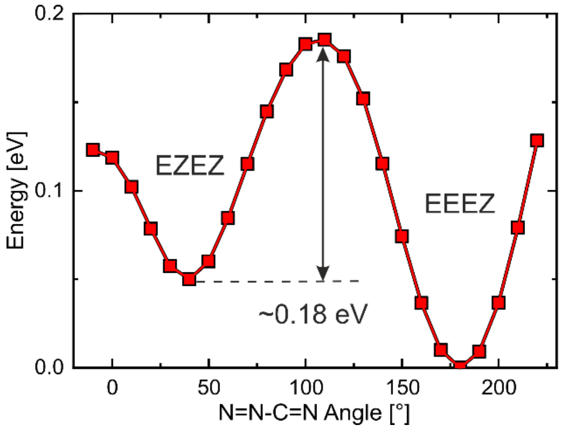

(S): -

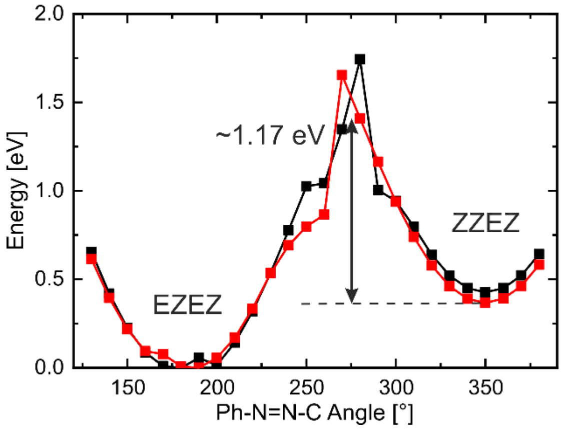

(T): Geom. Constraint BS: C-N=N 124.787°

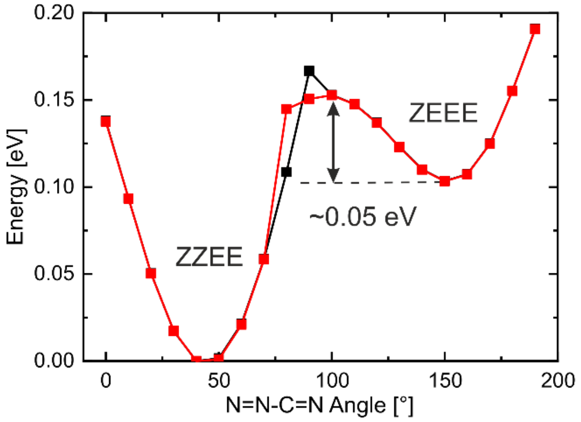

(U): -

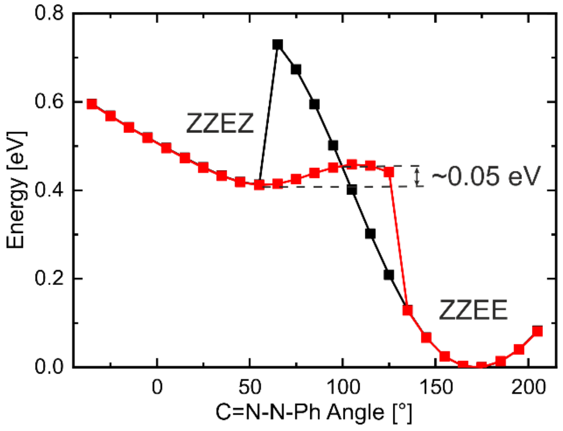

(V): -

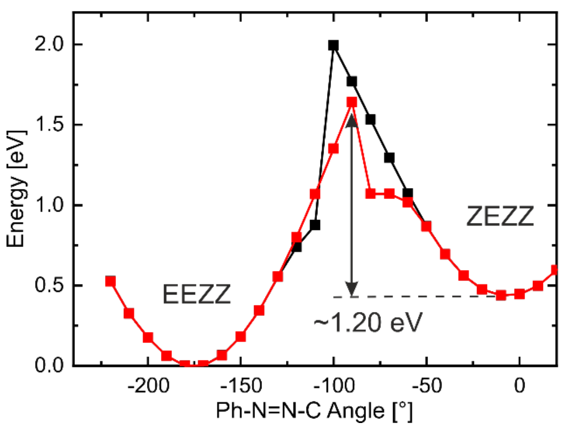

(W): Geom. Constraint: C-N=N 120°, C=N-N-C(Ph) 40° (both)

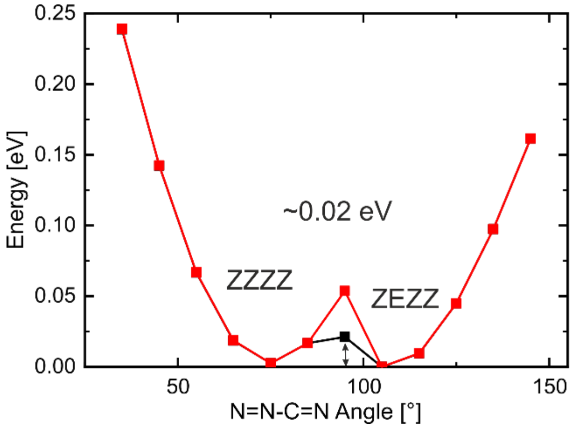

(X): Geom. Constraint: C=-N-N-C(Ph) 68.5° (both)

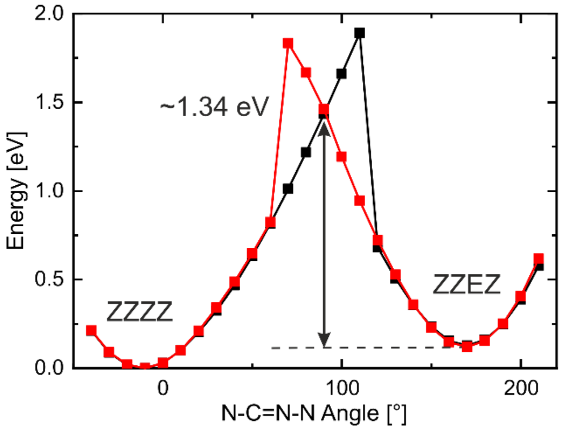

(Y): Geom. Constraint: N-C=N-N 8°/ BS: N-C=N-N 10°, C=N-N 122°, C-N=N 125°

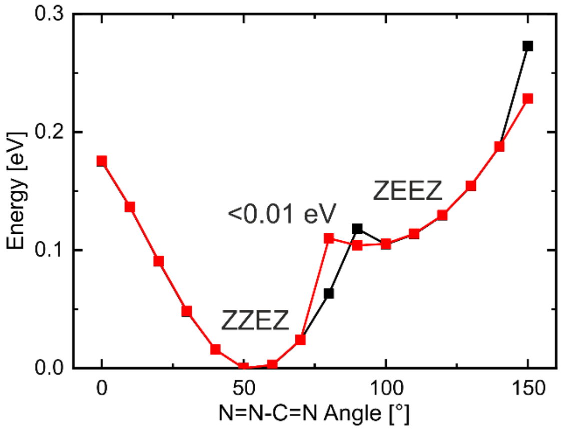

(Z): -

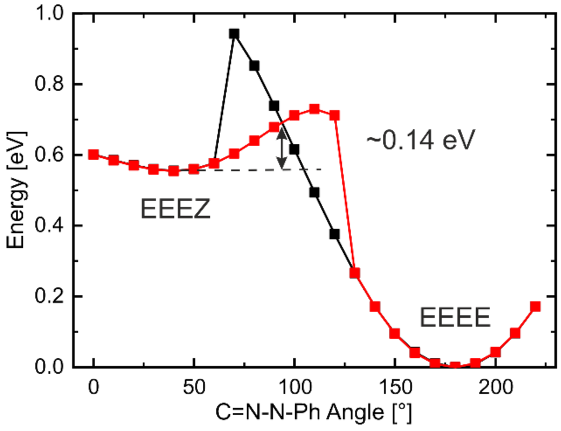

(AA): Geom. Constraint: C=N-N-C(Ph) 179.8° (both)

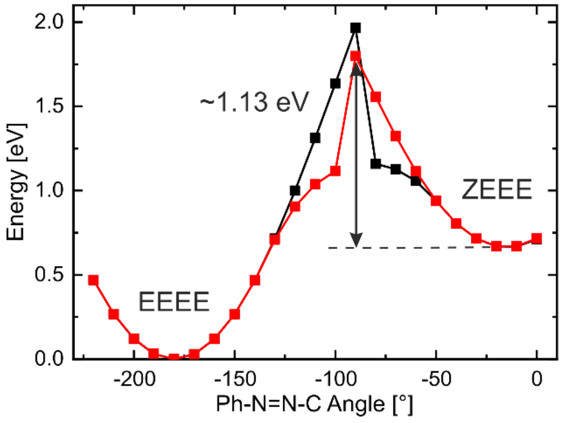

(AB): Geom. Constraint: N=N-C(Ph) 124° (both)

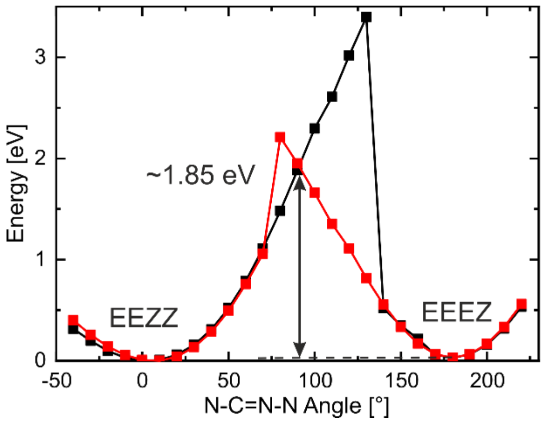

(AC): Geom. Constraint: C=N-N-C(Ph) 41.120° / BS: C=N-N-C(Ph) 40°, C=N-N 124.133°, N=C-N=N 180°

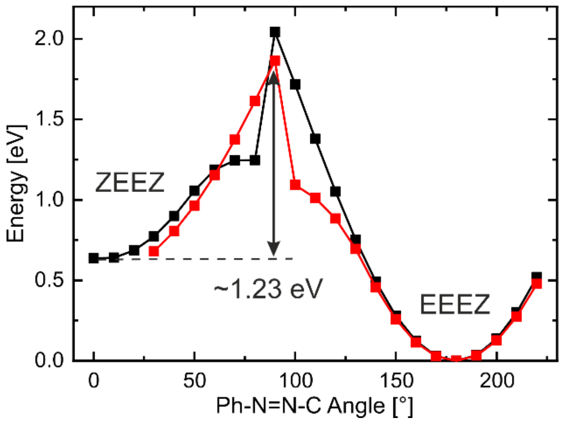

(AD): Geom. Constraint: C-N=N 120°/ BS: C-N=N 120°, N=N-C(Ph) 123°

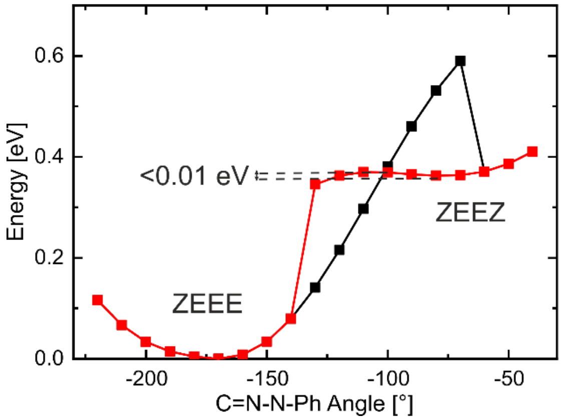

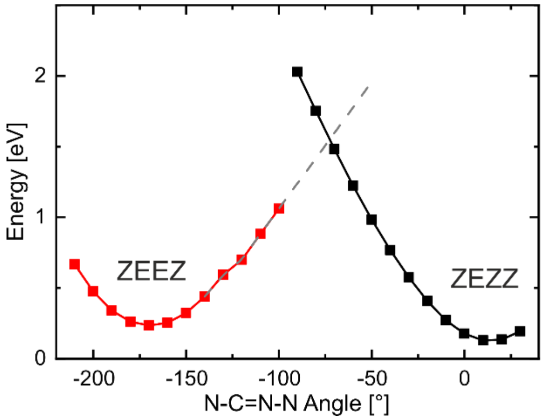

(AE): Geom. Constraint BS: N=C-N=N 93°

(AF): energy barrier only roughly estimated by extrapolation (gray dashed line) since the calculations did not converge; Geom. Constraint: N=C-N=N 254°, C=N-N 120° (both)

Supplementary Figure 8. Relaxed surface scan along a fixed torsional angle to estimate the barrier between different isomeric species of TPF. The calculations were carried out with Orca 5.0.1 (except where stated otherwise) at the UKS/B3LYP/def2-TZVP level of theory by optimizing all structural parameters except of the fixed torsional angle. The rotation was monitored in 10° steps from the energetically more stable to the energetically more unfavorable isomeric species (black) and vice versa (backscan [BS] red). Variations of the here described parameters are mentioned in the sub-caption of each individual scan.

Supplementary Table 1. XYZ-coordinates obtained after ground-state geometry optimization of the individual TPF isomers calculated with Orca 4.2.1 (RHF/B3LYP/def2-TZVP).

EEZE				EZEE			
N	-0.983354000	0.806534000	-3.868136000	N	-0.014382000	2.076873000	-3.464727000
C	-0.011473000	0.544607000	-3.031620000	C	-0.275052000	0.994247000	-2.810595000
N	-2.168947000	1.157969000	-3.414855000	N	1.236860000	2.429911000	-3.726977000
N	-0.314606000	0.580154000	-1.674191000	N	-1.612115000	0.589544000	-2.640353000
N	0.640828000	0.472023000	-0.863354000	N	-2.472035000	1.497381000	-2.636650000
C	1.314624000	0.241324000	-3.622007000	C	0.725384000	0.028941000	-2.284248000
C	2.171716000	-0.730309000	-3.093101000	C	0.693421000	-1.310416000	-2.685321000
C	1.709426000	0.916946000	-4.782910000	C	1.704119000	0.435405000	-1.370780000
C	3.378897000	-1.020528000	-3.716009000	C	1.626903000	-2.215472000	-2.197750000
C	2.917394000	0.626995000	-5.401297000	C	2.634336000	-0.474391000	-0.878707000
C	3.758750000	-0.344073000	-4.869704000	C	2.600509000	-1.800454000	-1.293807000
H	1.893308000	-1.266112000	-2.198302000	H	-0.071090000	-1.635049000	-3.379072000
H	1.056179000	1.673219000	-5.197488000	H	1.717560000	1.459764000	-1.017092000
H	4.024965000	-1.782434000	-3.297177000	H	1.593091000	-3.248000000	-2.522549000
H	3.203253000	1.163689000	-6.297434000	H	3.377753000	-0.147714000	-0.161895000
H	4.703277000	-0.570362000	-5.348422000	H	3.324131000	-2.508811000	-0.910430000
C	-3.255966000	1.361956000	-4.262282000	C	1.531111000	3.503895000	-4.567113000
C	-4.464402000	1.789573000	-3.701316000	C	2.872944000	3.780778000	-4.850489000
C	-3.168886000	1.152224000	-5.641996000	C	0.527029000	4.306508000	-5.116701000
C	-5.569494000	2.005358000	-4.511497000	C	3.204079000	4.846318000	-5.675250000
C	-4.282952000	1.374804000	-6.438247000	C	0.876067000	5.368596000	-5.938976000
C	-5.487870000	1.800494000	-5.885100000	C	2.209462000	5.648683000	-6.225169000
H	-4.532110000	1.955752000	-2.631762000	H	3.653188000	3.158058000	-4.426612000
H	-2.236102000	0.813966000	-6.068661000	H	-0.506310000	4.086799000	-4.892011000
H	-6.498598000	2.337575000	-4.065339000	H	4.246291000	5.048712000	-5.889344000
H	-4.208452000	1.207819000	-7.505993000	H	0.092639000	5.985441000	-6.361642000
H	-6.351246000	1.968274000	-6.515620000	H	2.470013000	6.479156000	-6.868419000

C	0.289866000	0.480391000	0.501863000
C	1.362008000	0.565691000	1.395308000
C	-1.017678000	0.382339000	0.998359000
C	1.134825000	0.573719000	2.764541000
C	-1.235821000	0.382765000	2.365698000
C	-0.163996000	0.482287000	3.253458000
H	2.363376000	0.631253000	0.989665000
H	-1.842481000	0.289578000	0.306529000
H	1.969996000	0.646058000	3.449646000
H	-2.245572000	0.299022000	2.748701000
H	-0.344140000	0.480107000	4.321446000
H	-2.298804000	1.222213000	-2.410241000

EZEZ

N	0.103149000	1.611738000	-3.392929000
C	-0.579872000	2.345371000	-2.594263000
N	1.377626000	1.892653000	-3.792083000
N	-1.863156000	1.840256000	-2.209888000
N	-2.495969000	1.237486000	-3.093239000
C	-0.287291000	3.661245000	-1.979839000
C	-0.751562000	3.963955000	-0.693889000
C	0.387581000	4.652478000	-2.701769000
C	-0.527301000	5.216834000	-0.138509000
C	0.593793000	5.908893000	-2.149704000
C	0.144120000	6.194201000	-0.864571000
H	-1.286479000	3.209801000	-0.134088000
H	0.740496000	4.440669000	-3.701415000
H	-0.881467000	5.430902000	0.862111000
H	1.110749000	6.667248000	-2.724395000
H	0.315418000	7.172571000	-0.433175000
C	2.489859000	2.228075000	-2.982627000

C	-3.812437000	1.060706000	-2.511988000
C	-4.774460000	2.072622000	-2.528509000
C	-4.210836000	-0.274123000	-2.360832000
C	-6.121416000	1.761363000	-2.394528000
C	-5.554941000	-0.576443000	-2.221605000
C	-6.514332000	0.436889000	-2.237906000
H	-4.440145000	3.095412000	-2.646576000
H	-3.458587000	-1.049969000	-2.346114000
H	-6.862283000	2.550871000	-2.408482000
H	-5.863711000	-1.607330000	-2.097254000
H	-7.563135000	0.190435000	-2.126937000
H	2.005361000	1.848494000	-3.409942000

EZZE

N	-0.138210000	1.907458000	-3.784040000
C	-0.469326000	0.951707000	-2.945592000
N	0.038306000	3.142111000	-3.375521000
N	-0.614421000	1.027817000	-1.571163000
N	-0.510303000	2.164432000	-1.018189000
C	-0.685023000	-0.397802000	-3.524366000
C	-0.606780000	-1.545576000	-2.727208000
C	-0.973667000	-0.552517000	-4.885789000
C	-0.804382000	-2.806082000	-3.277067000
C	-1.171396000	-1.812772000	-5.431550000
C	-1.087467000	-2.947680000	-4.630487000
H	-0.382999000	-1.444054000	-1.675133000
H	-1.050225000	0.326596000	-5.510561000
H	-0.734005000	-3.681946000	-2.643570000
H	-1.401356000	-1.908639000	-6.485777000
H	-1.245515000	-3.930753000	-5.056155000
C	0.491700000	4.136659000	-4.243898000

C	3.682151000	2.536850000	-3.649151000
C	2.476698000	2.210815000	-1.587634000
C	4.836039000	2.815875000	-2.933801000
C	3.638366000	2.500742000	-0.880291000
C	4.821626000	2.805038000	-1.541527000
H	3.692512000	2.560344000	-4.733159000
H	1.574874000	1.955526000	-1.052996000
H	5.748807000	3.051847000	-3.467012000
H	3.612730000	2.480253000	0.202483000
H	5.720293000	3.030823000	-0.982313000
C	-3.743004000	0.691787000	-2.690136000
C	-4.427868000	-0.017054000	-3.677677000
C	-4.300302000	0.825008000	-1.413062000
C	-5.659785000	-0.593966000	-3.396729000
C	-5.531006000	0.251682000	-1.140813000
C	-6.213735000	-0.459258000	-2.128933000
H	-3.972434000	-0.104203000	-4.655723000
H	-3.762253000	1.378725000	-0.656708000
H	-6.186839000	-1.145034000	-4.165422000
H	-5.966960000	0.357095000	-0.154810000
H	-7.176049000	-0.903215000	-1.907231000
H	1.628389000	1.237060000	-4.516745000

EZZZ

N	-0.146415000	1.816640000	-3.075038000
C	-1.146304000	1.583713000	-3.839839000
N	0.078862000	3.080054000	-2.564791000
N	-2.163334000	2.548202000	-4.128537000
N	-1.886459000	3.292462000	-5.085605000
C	-1.337435000	0.232440000	-4.404882000
C	-2.491587000	-0.090284000	-5.127075000

C	0.601816000	5.440794000	-3.749110000
C	0.839573000	3.865051000	-5.570449000
C	1.058735000	6.458806000	-4.572980000
C	1.291598000	4.894761000	-6.382518000
C	1.406386000	6.193853000	-5.893860000
H	0.326420000	5.650795000	-2.722475000
H	0.758229000	2.854347000	-5.942555000
H	1.143084000	7.464248000	-4.179939000
H	1.562401000	4.678127000	-7.408424000
H	1.764007000	6.989432000	-6.534397000
C	-0.645103000	2.169124000	0.387034000
C	-0.500042000	3.409275000	1.015144000
C	-0.912075000	1.030372000	1.159153000
C	-0.613513000	3.514945000	2.394363000
C	-1.024909000	1.144187000	2.534269000
C	-0.876716000	2.383173000	3.158333000
H	-0.295117000	4.281525000	0.407487000
H	-1.031272000	0.076547000	0.666214000
H	-0.497384000	4.479347000	2.872703000
H	-1.232304000	0.263134000	3.129075000
H	-0.968646000	2.462990000	4.234461000
H	-0.112829000	3.331749000	-2.380953000

ZEZE

N	-1.493107000	-1.560718000	-2.923551000
C	-0.997174000	-1.363333000	-1.744479000
N	-1.606363000	-0.514947000	-3.746416000
N	-0.824221000	-0.016424000	-1.307875000
N	0.176028000	0.435912000	-0.719563000
C	-0.871177000	-2.505458000	-0.816274000
C	-0.945089000	-2.330342000	0.569818000

C	-0.351820000	-0.749660000	-4.234801000	C	-0.719879000	-3.803192000	-1.320764000
C	-2.652909000	-1.360342000	-5.669983000	C	-0.873910000	-3.422270000	1.425513000
C	-0.514677000	-2.010668000	-4.783716000	C	-0.648183000	-4.890246000	-0.462756000
C	-1.666608000	-2.323641000	-5.503612000	C	-0.723833000	-4.706348000	0.914962000
H	-3.269474000	0.648710000	-5.261365000	H	-1.079489000	-1.339944000	0.984516000
H	0.537283000	-0.503766000	-3.671756000	H	-0.660759000	-3.946443000	-2.391117000
H	-3.553662000	-1.594297000	-6.223678000	H	-0.941758000	-3.267364000	2.495429000
H	0.259397000	-2.756555000	-4.649961000	H	-0.526861000	-5.886110000	-0.871105000
H	-1.791422000	-3.311659000	-5.928574000	H	-0.665666000	-5.556510000	1.582902000
C	-0.800838000	3.779182000	-1.713367000	C	-2.263995000	-0.616590000	-4.970939000
C	-0.388756000	5.045854000	-1.274464000	C	-2.382653000	0.536692000	-5.755210000
C	-2.028030000	3.277336000	-1.275394000	C	-2.776432000	-1.827938000	-5.444806000
C	-1.188361000	5.791110000	-0.425013000	C	-2.996642000	0.474787000	-6.996893000
C	-2.827411000	4.041710000	-0.429479000	C	-3.383888000	-1.873701000	-6.692529000
C	-2.420726000	5.297350000	0.000366000	C	-3.499185000	-0.730657000	-7.477951000
H	0.558962000	5.443012000	-1.619254000	H	-1.986054000	1.476926000	-5.389172000
H	-2.352869000	2.292373000	-1.572690000	H	-2.696628000	-2.713876000	-4.832160000
H	-0.853176000	6.768583000	-0.101343000	H	-3.079445000	1.375493000	-7.592529000
H	-3.775510000	3.636721000	-0.097219000	H	-3.777736000	-2.816823000	-7.051209000
H	-3.049829000	5.884107000	0.657375000	H	-3.976258000	-0.776948000	-8.447948000
C	-2.850362000	4.295092000	-5.383814000	C	1.401796000	-0.271298000	-0.576160000
C	-2.618913000	5.013675000	-6.555899000	C	2.118862000	-0.022160000	0.596986000
C	-3.955209000	4.592225000	-4.579780000	C	1.977174000	-1.040939000	-1.592556000
C	-3.499147000	6.017041000	-6.939716000	C	3.366287000	-0.597283000	0.783961000
C	-4.823186000	5.600413000	-4.964167000	C	3.242813000	-1.579780000	-1.413000000
C	-4.599989000	6.311943000	-6.143394000	C	3.933389000	-1.374762000	-0.222163000
H	-1.747685000	4.768162000	-7.149536000	H	1.678152000	0.618790000	1.349869000
H	-4.106500000	4.039548000	-3.663436000	H	1.452880000	-1.190513000	-2.526434000
H	-3.323722000	6.571670000	-7.852587000	H	3.907861000	-0.421336000	1.704855000
H	-5.675790000	5.841665000	-4.341517000	H	3.693053000	-2.161112000	-2.207897000
H	-5.284027000	7.099642000	-6.434577000	H	4.918047000	-1.803382000	-0.087305000

H	1.033196000	3.121153000	-2.241607000	H	-1.430231000	0.411326000	-3.369924000
**ZEZZ**				**ZZEE**			
N	-1.232957000	-0.725132000	-2.271975000	N	0.089279000	-0.920702000	-2.723100000
C	-2.023699000	-1.427376000	-1.547062000	C	0.788964000	-1.492516000	-1.804955000
N	-1.537650000	0.583474000	-2.596273000	N	0.234542000	-1.244095000	-4.006450000
N	-3.379501000	-1.067857000	-1.244392000	N	0.371403000	-1.328540000	-0.451906000
N	-3.775545000	-0.647586000	-0.150259000	N	-0.041728000	-0.275023000	0.051638000
C	-1.625498000	-2.805329000	-1.170226000	C	1.866102000	-2.497053000	-1.976430000
C	-2.539989000	-3.698808000	-0.602479000	C	1.881646000	-3.664420000	-1.202219000
C	-0.307545000	-3.233070000	-1.380163000	C	2.916222000	-2.274095000	-2.877055000
C	-2.144650000	-4.983806000	-0.247708000	C	2.910527000	-4.586785000	-1.340165000
C	0.081951000	-4.512808000	-1.019922000	C	3.945694000	-3.198414000	-3.008372000
C	-0.834411000	-5.395883000	-0.451973000	C	3.944276000	-4.359488000	-2.243209000
H	-3.567302000	-3.399640000	-0.447527000	H	1.085652000	-3.834125000	-0.490525000
H	0.398787000	-2.547446000	-1.826045000	H	2.946994000	-1.357152000	-3.453109000
H	-2.867543000	-5.663492000	0.186039000	H	2.906568000	-5.486909000	-0.738001000
H	1.105781000	-4.825923000	-1.182973000	H	4.755084000	-3.004256000	-3.701326000
H	-0.527191000	-6.396214000	-0.174112000	H	4.746899000	-5.079330000	-2.345608000
C	-2.680238000	0.977824000	-3.348458000	C	-0.574565000	-0.684839000	-4.995539000
C	-2.884100000	2.351892000	-3.517387000	C	-0.517242000	-1.218691000	-6.287546000
C	-3.561650000	0.077250000	-3.943962000	C	-1.408253000	0.406511000	-4.734445000
C	-3.949314000	2.814120000	-4.274191000	C	-1.279843000	-0.662920000	-7.304684000
C	-4.635251000	0.552789000	-4.690775000	C	-2.167165000	0.947669000	-5.762686000
C	-4.837174000	1.915715000	-4.861468000	C	-2.109834000	0.424491000	-7.051370000
H	-2.206676000	3.051206000	-3.041413000	H	0.121710000	-2.071200000	-6.490227000
H	-3.408851000	-0.985764000	-3.836649000	H	-1.442735000	0.822092000	-3.737996000
H	-4.094872000	3.880743000	-4.395119000	H	-1.226706000	-1.086758000	-8.299891000
H	-5.314148000	-0.156839000	-5.147291000	H	-2.806718000	1.796330000	-5.552768000
H	-5.675374000	2.277024000	-5.443392000	H	-2.702107000	0.858194000	-7.846390000
C	-2.898840000	-0.361062000	0.941081000	C	0.052349000	1.007280000	-0.570913000

C	-3.347306000	-0.766954000	2.199607000
C	-1.724416000	0.383813000	0.819036000
C	-2.583865000	-0.496780000	3.325042000
C	-0.986035000	0.680147000	1.958424000
C	-1.401879000	0.229892000	3.207068000
H	-4.286804000	-1.299103000	2.273659000
H	-1.421789000	0.764900000	-0.146843000
H	-2.919218000	-0.836207000	4.296743000
H	-0.082171000	1.269927000	1.868988000
H	-0.817491000	0.460606000	4.088719000
H	-0.698327000	1.005167000	-2.966948000

ZZEZ

N	-0.086557000	-1.083294000	-3.982611000
C	0.406838000	0.011194000	-4.423290000
N	-0.107895000	-2.241384000	-4.729029000
N	0.126595000	1.169659000	-3.606848000
N	0.366939000	1.254889000	-2.399596000
C	1.122427000	0.345731000	-5.673667000
C	1.997238000	-0.576611000	-6.262284000
C	1.005866000	1.622951000	-6.237810000
C	2.734099000	-0.228065000	-7.384467000
C	1.734890000	1.959779000	-7.370859000
C	2.601035000	1.037705000	-7.947218000
H	2.100876000	-1.564222000	-5.835628000
H	0.339418000	2.343340000	-5.784746000
H	3.409616000	-0.950095000	-7.826151000
H	1.625908000	2.946693000	-7.802923000
H	3.168147000	1.301300000	-8.831163000
C	-0.692970000	-2.389545000	-6.011569000
C	-0.596204000	-3.654121000	-6.605195000

C	1.265825000	1.527488000	-1.026119000
C	-1.079808000	1.820282000	-0.529483000
C	1.328697000	2.840344000	-1.470894000
C	-1.015280000	3.123162000	-1.005537000
C	0.187856000	3.639004000	-1.477272000
H	2.155093000	0.909877000	-1.016276000
H	-2.000755000	1.416695000	-0.128824000
H	2.273321000	3.243271000	-1.815027000
H	-1.903099000	3.743387000	-0.988480000
H	0.241618000	4.661056000	-1.829440000
H	0.847269000	-2.008954000	-4.266037000

ZZZE

N	-1.346569000	-0.764423000	-3.716473000
C	-0.243354000	-0.738458000	-3.055806000
N	-1.431088000	-0.218516000	-4.936507000
N	0.990648000	-0.317995000	-3.672004000
N	1.282307000	0.860832000	-3.926441000
C	-0.132788000	-1.402565000	-1.748448000
C	-1.258087000	-1.958890000	-1.124411000
C	1.101851000	-1.470438000	-1.091127000
C	-1.146347000	-2.560549000	0.118483000
C	1.207848000	-2.077714000	0.154727000
C	0.086663000	-2.623624000	0.766034000
H	-2.214551000	-1.911557000	-1.626064000
H	1.985062000	-1.059491000	-1.562725000
H	-2.025775000	-2.984863000	0.587148000
H	2.171998000	-2.122520000	0.645828000
H	0.168611000	-3.093608000	1.737663000
C	-2.581309000	-0.349518000	-5.711478000
C	-2.553610000	0.119130000	-7.030478000

C	-1.373082000	-1.374462000	-6.684581000
C	-1.169774000	-3.895578000	-7.843450000
C	-1.938001000	-1.626291000	-7.930289000
C	-1.840839000	-2.880089000	-8.519805000
H	-0.060741000	-4.442838000	-6.088924000
H	-1.477787000	-0.397130000	-6.238800000
H	-1.082450000	-4.879930000	-8.286962000
H	-2.464227000	-0.827684000	-8.438668000
H	-2.278582000	-3.064654000	-9.491862000
C	1.150266000	0.300818000	-1.676765000
C	2.421025000	-0.092880000	-2.097603000
C	0.676326000	-0.089223000	-0.425150000
C	3.195967000	-0.901551000	-1.277171000
C	1.443229000	-0.925593000	0.373507000
C	2.705809000	-1.332966000	-0.048090000
H	2.807910000	0.249301000	-3.048501000
H	-0.293537000	0.262016000	-0.098117000
H	4.187747000	-1.194055000	-1.598404000
H	1.062958000	-1.244698000	1.335616000
H	3.312705000	-1.966678000	0.586407000
H	-0.376268000	-2.994191000	-4.113047000

ZZZZ

N	-1.515012000	-0.833694000	-3.900119000
C	-2.176085000	-0.433590000	-2.878536000
N	-1.752399000	-0.217012000	-5.133006000
N	-2.982783000	0.754842000	-2.862135000
N	-4.087978000	0.868018000	-3.403100000
C	-1.945915000	-1.064050000	-1.560431000
C	-1.213412000	-2.254190000	-1.464684000
C	-2.474109000	-0.496585000	-0.396142000

C	-3.756915000	-0.915237000	-5.209176000
C	-3.681656000	0.019600000	-7.831242000
C	-4.877434000	-1.007220000	-6.023711000
C	-4.853537000	-0.544077000	-7.335574000
H	-1.644053000	0.557717000	-7.426345000
H	-3.778380000	-1.278260000	-4.192778000
H	-3.641893000	0.383330000	-8.850463000
H	-5.781957000	-1.449782000	-5.624310000
H	-5.732095000	-0.624093000	-7.962450000
C	0.517900000	1.980645000	-3.472152000
C	-0.015330000	2.095983000	-2.185738000
C	0.438139000	3.054556000	-4.361488000
C	-0.654486000	3.271351000	-1.815151000
C	-0.235394000	4.210642000	-3.994913000
C	-0.780970000	4.321929000	-2.719446000
H	0.096077000	1.293116000	-1.471431000
H	0.906622000	2.963819000	-5.333179000
H	-1.054498000	3.366590000	-0.813785000
H	-0.317217000	5.032064000	-4.695497000
H	-1.287223000	5.231954000	-2.423766000
H	-0.598514000	0.131040000	-5.394006000

EEEE

N	-0.058220000	2.646094000	-3.368202000
C	-0.518924000	1.447146000	-3.178690000
N	1.226982000	2.819547000	-3.621292000
N	-1.890479000	1.419382000	-2.942325000
N	-2.374324000	0.273666000	-2.756123000
C	0.320645000	0.213597000	-3.213777000
C	0.320963000	-0.617114000	-4.336748000
C	1.131225000	-0.118442000	-2.125446000

C	-1.023439000	-2.861085000	-0.233563000
C	-2.276273000	-1.107908000	0.836863000
C	-1.552607000	-2.290270000	0.922542000
H	-0.801262000	-2.688545000	-2.364668000
H	-3.024379000	0.432838000	-0.453834000
H	-0.459716000	-3.783655000	-0.172244000
H	-2.685573000	-0.654206000	1.730632000
H	-1.398505000	-2.765425000	1.883267000
C	-1.259428000	1.080292000	-5.416435000
C	-1.389148000	1.538841000	-6.734360000
C	-0.663885000	1.908687000	-4.465614000
C	-0.929256000	2.796136000	-7.089285000
C	-0.213192000	3.173347000	-4.833395000
C	-0.339700000	3.627611000	-6.138802000
H	-1.862125000	0.902524000	-7.473936000
H	-0.539836000	1.573253000	-3.447463000
H	-1.039445000	3.132548000	-8.113028000
H	0.246621000	3.804221000	-4.082469000
H	0.012948000	4.612519000	-6.415217000
C	-4.777661000	-0.225815000	-4.008942000
C	-4.911423000	-1.484169000	-3.420388000
C	-5.440343000	0.064222000	-5.202536000
C	-5.674363000	-2.457077000	-4.053714000
C	-6.169739000	-0.924359000	-5.845443000
C	-6.290046000	-2.187388000	-5.271504000
H	-4.455031000	-1.694815000	-2.463695000
H	-5.356405000	1.060969000	-5.615806000
H	-5.789875000	-3.429228000	-3.590985000
H	-6.659431000	-0.705960000	-6.785810000
H	-6.877209000	-2.953187000	-5.762636000
H	-1.467881000	-0.870354000	-5.848887000

C	1.115093000	-1.756763000	-4.369251000
C	1.924726000	-1.260459000	-2.158307000
C	1.917953000	-2.081167000	-3.280295000
H	-0.310548000	-0.372562000	-5.181614000
H	1.129739000	0.513753000	-1.245408000
H	1.103023000	-2.395013000	-5.244037000
H	2.542461000	-1.511005000	-1.304836000
H	2.533554000	-2.971703000	-3.305529000
C	1.769591000	4.083843000	-3.854933000
C	3.139063000	4.179555000	-4.122369000
C	0.985347000	5.240438000	-3.827469000
C	3.715848000	5.418631000	-4.359807000
C	1.578354000	6.471794000	-4.067185000
C	2.940822000	6.573602000	-4.334521000
H	3.748013000	3.282486000	-4.144013000
H	-0.071622000	5.158570000	-3.620675000
H	4.777214000	5.479612000	-4.565884000
H	0.964833000	7.364122000	-4.044951000
H	3.392034000	7.539391000	-4.520969000
C	-3.767469000	0.242327000	-2.523386000
C	-4.313257000	-1.024947000	-2.303901000
C	-4.600894000	1.369207000	-2.505180000
C	-5.673749000	-1.171065000	-2.067809000
C	-5.956959000	1.215121000	-2.269675000
C	-6.499130000	-0.052033000	-2.050812000
H	-3.649419000	-1.879857000	-2.322776000
H	-4.169002000	2.345010000	-2.677381000
H	-6.089880000	-2.156248000	-1.897777000
H	-6.601359000	2.085843000	-2.256224000
H	-7.560869000	-0.162562000	-1.868246000
H	1.844936000	2.015681000	-3.668846000

EEEZ

N	-0.022373000	2.217071000	-4.163070000
C	-0.539013000	2.182853000	-2.974794000
N	1.186032000	2.730651000	-4.444840000
N	-1.813474000	1.581226000	-3.019608000
N	-2.414797000	1.487174000	-1.925184000
C	-0.006639000	2.795827000	-1.727505000
C	0.157497000	2.061736000	-0.550338000
C	0.284371000	4.161959000	-1.715492000
C	0.622172000	2.676560000	0.604525000
C	0.738190000	4.779106000	-0.556418000
C	0.912683000	4.036859000	0.605795000
H	-0.080948000	1.007096000	-0.538062000
H	0.150106000	4.744680000	-2.618137000
H	0.749417000	2.093881000	1.508593000
H	0.954824000	5.840169000	-0.562144000
H	1.270076000	4.516038000	1.508893000
C	2.411659000	2.625404000	-3.733795000
C	3.416890000	3.542260000	-4.050448000
C	2.676139000	1.603702000	-2.822401000
C	4.668797000	3.439404000	-3.460692000
C	3.928593000	1.516399000	-2.228103000
C	4.929836000	2.429246000	-2.540439000
H	3.206423000	4.339368000	-4.753736000
H	1.916004000	0.871848000	-2.592322000
H	5.438136000	4.158543000	-3.713115000
H	4.123847000	0.719285000	-1.521565000
H	5.903035000	2.353949000	-2.072813000
C	-3.696282000	0.888800000	-1.997512000
C	-4.387744000	0.796673000	-0.787855000

EEZZ

N	-0.330915000	1.600837000	-2.742943000
C	-1.201981000	1.617978000	-3.703577000
N	-0.040244000	2.618836000	-1.923861000
N	-1.904308000	2.792167000	-4.003615000
N	-2.504881000	2.799210000	-5.106869000
C	-1.315187000	0.361296000	-4.497741000
C	-2.556342000	-0.154215000	-4.882287000
C	-0.161659000	-0.352117000	-4.834384000
C	-2.638729000	-1.353352000	-5.578466000
C	-0.244226000	-1.545764000	-5.539350000
C	-1.484120000	-2.052240000	-5.913827000
H	-3.462521000	0.379674000	-4.633313000
H	0.802073000	0.036614000	-4.533657000
H	-3.609595000	-1.741877000	-5.860789000
H	0.661842000	-2.080299000	-5.797519000
H	-1.550431000	-2.983514000	-6.462782000
C	-0.857909000	3.605194000	-1.316784000
C	-0.207298000	4.715443000	-0.773174000
C	-2.236308000	3.468755000	-1.145542000
C	-0.924083000	5.679214000	-0.075730000
C	-2.944699000	4.438939000	-0.450766000
C	-2.298625000	5.550133000	0.085153000
H	0.862285000	4.823880000	-0.910266000
H	-2.751149000	2.608610000	-1.546276000
H	-0.404809000	6.536745000	0.333833000
H	-4.013590000	4.320569000	-0.322029000
H	-2.860698000	6.302936000	0.622600000
C	-3.155177000	4.007258000	-5.439236000
C	-3.737829000	4.040539000	-6.709333000

C	-4.282787000	0.401919000	-3.172952000
C	-5.653445000	0.226369000	-0.746564000
C	-5.544644000	-0.166862000	-3.123499000
C	-6.234412000	-0.256971000	-1.913666000
H	-3.913816000	1.181995000	0.105931000
H	-3.737070000	0.478944000	-4.102870000
H	-6.185358000	0.158957000	0.194240000
H	-5.999222000	-0.544503000	-4.031398000
H	-7.220971000	-0.702821000	-1.885011000
H	1.310022000	2.767175000	-5.444860000

ZEEE

N	1.031575000	0.608750000	-4.142738000
C	1.104048000	0.259755000	-2.899750000
N	0.566374000	-0.227830000	-5.058693000
N	1.742400000	1.252876000	-2.099107000
N	1.517206000	1.415484000	-0.886083000
C	0.839687000	-1.120168000	-2.396527000
C	-0.425876000	-1.709991000	-2.477251000
C	1.903400000	-1.877423000	-1.894774000
C	-0.622315000	-3.023177000	-2.065679000
C	1.704446000	-3.188325000	-1.480748000
C	0.441978000	-3.765594000	-1.566464000
H	-1.266502000	-1.130363000	-2.838409000
H	2.889629000	-1.434582000	-1.832187000
H	-1.610642000	-3.461733000	-2.125359000
H	2.538462000	-3.760706000	-1.094185000
H	0.287756000	-4.787523000	-1.243658000
C	0.506857000	0.122478000	-6.408115000
C	-0.008679000	-0.810335000	-7.314096000
C	0.942139000	1.368246000	-6.868757000

C	-3.249411000	5.126117000	-4.599442000
C	-4.403196000	5.178864000	-7.144029000
C	-3.918611000	6.255638000	-5.039472000
C	-4.495619000	6.287852000	-6.310134000
H	-3.649851000	3.163441000	-7.337472000
H	-2.800733000	5.090450000	-3.616957000
H	-4.849151000	5.200908000	-8.130402000
H	-3.994755000	7.120279000	-4.391543000
H	-5.016632000	7.176312000	-6.644931000
H	0.774626000	2.389609000	-1.376809000

ZEEZ

N	-1.383135000	-1.879400000	-3.868840000
C	-0.848879000	-0.758376000	-4.161967000
N	-1.041820000	-2.999958000	-4.651747000
N	-1.424146000	0.403889000	-3.519213000
N	-1.036152000	0.905430000	-2.461577000
C	0.158398000	-0.397896000	-5.190374000
C	1.293156000	-1.195562000	-5.376954000
C	0.026001000	0.783134000	-5.929679000
C	2.270353000	-0.816431000	-6.285403000
C	1.001847000	1.149272000	-6.847699000
C	2.126297000	0.352469000	-7.027044000
H	1.401341000	-2.108565000	-4.809892000
H	-0.845769000	1.407366000	-5.788115000
H	3.147404000	-1.437733000	-6.416987000
H	0.882790000	2.059596000	-7.421768000
H	2.887700000	0.639359000	-7.741664000
C	-1.569510000	-3.160396000	-5.957533000
C	-1.341090000	-4.389808000	-6.589927000
C	-2.285938000	-2.174632000	-6.636408000

C	-0.086203000	-0.499706000	-8.663906000
C	0.855167000	1.663544000	-8.222008000
C	0.343681000	0.739122000	-9.128486000
H	-0.346249000	-1.777607000	-6.958223000
H	1.341498000	2.083249000	-6.164499000
H	-0.485871000	-1.231439000	-9.355017000
H	1.194951000	2.630863000	-8.571376000
H	0.282061000	0.980299000	-10.181624000
C	0.403758000	0.856469000	-0.198407000
C	0.621173000	0.236734000	1.033771000
C	-0.902523000	1.106381000	-0.628927000
C	-0.457667000	-0.193052000	1.791912000
C	-1.976477000	0.705272000	0.155187000
C	-1.760569000	0.040650000	1.357739000
H	1.637261000	0.093753000	1.378746000
H	-1.067024000	1.637670000	-1.557705000
H	-0.283165000	-0.696348000	2.734844000
H	-2.986661000	0.915290000	-0.174732000
H	-2.600574000	-0.275415000	1.963117000
H	0.276185000	-1.162660000	-4.793510000
C	-1.819962000	-4.623876000	-7.868747000
C	-2.755926000	-2.419290000	-7.923399000
C	-2.528957000	-3.637006000	-8.550056000
H	-0.776472000	-5.156492000	-6.071243000
H	-2.497619000	-1.227704000	-6.163206000
H	-1.631935000	-5.581394000	-8.339106000
H	-3.313580000	-1.643398000	-8.433817000
H	-2.897411000	-3.817629000	-9.551476000
C	0.017212000	0.362741000	-1.660202000
C	0.966117000	1.287419000	-1.217487000
C	0.062278000	-0.958393000	-1.210261000
C	2.000143000	0.875895000	-0.390787000
C	1.080434000	-1.348984000	-0.347678000
C	2.057653000	-0.443695000	0.050193000
H	0.881586000	2.318033000	-1.537443000
H	-0.704199000	-1.655029000	-1.514029000
H	2.749459000	1.589433000	-0.071970000
H	1.107592000	-2.369112000	0.014949000
H	2.851353000	-0.759269000	0.715564000
H	-1.247389000	-3.814519000	-4.090953000
